# Global Reprogramming of Gene Transcription in *Trichoderma reesei* by Overexpressing an Artificial Transcription Factor for Improved Cellulase Production and Identification of Ypr1 as an Associated Regulator

**DOI:** 10.3389/fbioe.2020.00649

**Published:** 2020-07-03

**Authors:** Fei Zhang, Jia-Xiang Li, Verawat Champreda, Chen-Guang Liu, Feng-Wu Bai, Xin-Qing Zhao

**Affiliations:** ^1^State Key Laboratory of Microbial Metabolism, Joint International Research Laboratory of Metabolic & Developmental Sciences, School of Life Sciences and Biotechnology, Shanghai Jiao Tong University, Shanghai, China; ^2^Biorefinery and Bioproduct Research Group, Enzyme Technology Laboratory, National Center for Genetic Engineering and Biotechnology, Pathum Thani, Thailand

**Keywords:** *Trichoderma reesei*, cellulase production, artificial zinc finger protein, comparative transcriptome analysis, transcription factor, lignocellulosic biomass

## Abstract

Synthetic biology studies on filamentous fungi are providing unprecedented opportunities for optimizing this important category of microbial cell factory. Artificial transcription factor can be designed and used to offer novel modes of regulation on gene transcription network. *Trichoderma reesei* is commonly used for cellulase production. In our previous studies, a plasmid library harboring genes encoding artificial zinc finger proteins (AZFPs) was constructed for engineering *T. reesei*, and the mutant strains with improved cellulase production were selected. However, the underlying mechanism by which AZFP function remain unclear. In this study, a *T. reesei* Rut-C30 mutant strain *T. reesei* U5 bearing an AZFP named as AZFP-U5 was focused, which secretes high level protein and shows significantly improved cellulase and xylanase production comparing with its parental strain. In addition, enhanced sugar release was achieved from lignocellulosic biomass using the crude cellulase from *T. reesei* U5. Comparative transcriptome analysis was further performed, which showed reprogramming of global gene transcription and elevated transcription of genes encoding glycoside hydrolases by overexpressing *AZFP-U5*. Furthermore, 15 candidate regulatory genes which showed remarkable higher transcription levels by *AZFP-U5* insertion were overexpressed in *T. reesei* Rut-C30 to examine their effects on cellulase biosynthesis. Among these genes, *TrC30_93861* (*ypr1*) and *TrC30_74374* showed stimulating effects on filter paper activity (FPase), but deletion of these two genes did not affect cellulase activity. In addition, increased yellow pigment production in *T. reesei* Rut-C30 by overexpression of gene *ypr1* was observed, and changes of cellulase gene transcription were revealed in the *ypr1* deletion mutant, suggesting possible interaction between pigment production and cellulase gene transcription. The results in this study revealed novel aspects in regulation of cellulase gene expression by the artificial regulators. In addition, the candidate genes and processes identified in the transcriptome data can be further explored for synthetic biology design and metabolic engineering of *T. reesei* to enhance cellulase production.

## Introduction

Cellulase is widely used in detergents, textiles, pulp processing, food, and feed industries (Sharma et al., 2016). The application of cellulase in lignocellulosic biorefinery for producing renewable biofuels and biochemicals has particularly received widespread interest owning to the gradual depletion of fossil fuels and environmental concerns (Tiwari et al., 2018). Efficient production of cellulases is essential for reducing the bioconversion cost of lignocellulosic biomass. *Trichoderma reesei* (an anamorph of *Hypocrea jecorina*) and its derivative strains have been widely used for cellulase production (Bischof et al., 2016). However, so far high cellulase production cost is still a bottleneck for practical applications.

The *T. reesei* genome commonly contains over 220 putative genes encoding candidate carbohydrate-active enzymes (CAZymes), which include cellulases such as cellobiohydrolases (CBH), endoglucanases (EG), and β-glucosidases (BGL), as well as hemicellulases. In addition, auxiliary proteins that hydrolyze lignocellulose synergistically are also important for biodegradation of cellulosic feedstocks (Hakkinen et al., 2012). Cellulase biosynthesis by *T. reesei* is induced by various inducers such as cellulose, sophorose, lactose, and cellobiose, but is repressed by glucose. Besides these carbon sources, cellulase production is also responsive to a variety of environmental and physiological factors, such as Ca^2+^, light, pH, and physiological state of the mitochondria (Abrahao Neto et al., 1995; He et al., 2014; Chen et al., 2016; Schmoll, 2018). On the other hand, transcription factors also play important roles in cellulase production. So far, several key regulatory factors were well-characterized. Transcription factor Xyr1 is the major positive regulator governing a complex regulatory network for expression of (hemi-) cellulase genes, and recently, the function of Ace3 is characterized for cellulase production, and its cross talk with Xyr1 and Crt1 was revealed (Zhang et al., 2019). Furthermore, other regulators, such as Ace2, BglR, and Vib1 have also been identified to regulate cellulase production (Aro et al., 2001; Nitta et al., 2012; Zhang et al., 2018a). Recently, our group identified a novel repressor Ctf1 for cellulase biosynthesis in *T. reesei* Rut-C30 (Meng et al., 2020). In addition, transcription factor Cre1 mediates carbon catabolite repression (CCR) to suppress cellulase expression (Nakari-Setala et al., 2009). However, the detailed regulatory mechanisms of cellulase production from *T. reesei* are known to be complicated and are still not fully clear.

Synthetic biology studies of *T. reesei* are still in its early stage, but have opened new opportunities for metabolic engineering of this important species (Druzhinina and Kubicek, 2017; Liu and Qu, 2019). Artificial transcription factors (ATFs) could perturb and remodel the innate regulatory network, leading to new phenotypes (Yang et al., 2019). Several chimeric transcription factors were reported to have positive effects on cellulase production for *T. reesei*, which usually comprised DNA binding domains from characterized regulators with different effecter domains, such as minimal transcriptional activators comprising DBDace2(cre1)-linker-VP16AD, regulators DBDxyr1-EDypr1, and Xyr1-DBDcre1 (Zhang et al., 2017, 2018b; Derntl et al., 2019). Also, some novel fused regulators containing DBDcre1 and activation domains of Ace3, Clr2, Ace2, or Xyr1 were found to regulate the cellulase production profile in *T. reesei*, especially for CBH1 (Wang et al., 2019).

In our previous studies, the artificial zinc finger protein (AZFP) AZFP-U3 containing Cyc2-His2 type DNA binding domain significantly enhanced cellulase production in *T. reesei* Rut-C30 (Zhang et al., 2016). In our recent report, AZFP-M2 also led to improved cellulase activity in *T. reesei* Rut-C30 (Meng et al., 2020), but these two AZFPs showed different stimulating patterns on cellulase compositions. It will be of great interest to test more AZFPs for their regulatory functions, and reveal how the ATFs regulate cellulase production in *T. reesei*.

In this study, an additional AZFP which we named AZFP-U5 was identified to have a positive effect on cellulase production in *T. reesei* Rut-C30, whose feature on the regulatory network was further analyzed by comparative transcriptome analysis. Meanwhile, candidate regulatory genes related to cellulase production were further explored. The results in this study would provide novel insights in synthetic biology design and metabolic engineering of *T. reesei* for cellulase production.

## Materials and Methods

### Strains and Culture Media

*T. reesei* Rut-C30 was obtained from ARS Culture Collection (NRRL, Peoria, IL). Conidial suspensions were prepared by cultivating the strain on malt extract agar (MEA) medium (20 g/L Malt extract, 20 g/L Agar) at 28°C for 7 days. After harvesting, the conidia were suspended in 20% glycerol, which were then gauze-filtered, and stored at −80°C before use. Mandels-Andreotti (MA) medium (Mandels and Andreotti, 1978) containing 2% (m/v) microcrystalline cellulose (Merck, Germany) and 2% (m/v) wheat bran was used as a cellulase production medium. To induce cellulase production in the presence of soluble carbon sources, the carbon source in MA medium was replaced with 2% (m/v) glucose or 2% (m/v) lactose.

For cellulase production, the conidia of *Trichoderma* strains were collected from MEA medium, and cultured in 50 ml MA medium in a 250 mL Erlenmeyer flask supplemented with 0.1% (w/v) peptone and 2% (w/v) glucose to induce spore germination. After pre-cultivation at 28°C and 150 rpm in a rotary shaker for 24 h, 5 mL mycelia were taken from the germination medium and were added into 45 mL cellulase production medium in a 250 mL flask (10% inoculation size).

*Escherichia coli* DH5α was used for plasmid propagation and DNA manipulation. *Agrobacterium tumefaciens* AGL-1 was used for the *A. tumefaciens*-mediated genetic transformation (ATMT) of *T. reesei*. Transformation of *A. tumefaciens* AGL-1 with the pCB303-ZFP library and screening of *T. reesei* mutants were performed using the methods descried previously (Zhang et al., 2016). The *T. reesei* U5 mutant strain selected from the mutants was preserved in China General Microbiological Culture Collection Center (CGMCC) with the accession number of CGMCC 10649.

### Determination of Cellulase Activity, Protein Concentration, and Sporulation Assay

The *T. reesei* cultured sample (1 mL) was collected each day and centrifuged at 10,000 rpm for 5 min, from which the supernatant was used for cellulase activity measurement and protein concentration assay. Filter paper activity (FPase activity), endoglucanase (CMCase) activity, and β-glucosidase activity were measured according to the standard IUPAC procedures. The FPase activity is expressed as filter paper units, one unit of filter paper enzymatic activity is defined as the amount of enzyme that releases 1 μmol of glucose per minute from 50 mg filter paper stripe. The total xylanase activity was assayed with 1% beechwood xylan (Sigma-Aldrich, USA) as the substrate according to the standard protocol (Bailey et al., 1992). Concentration of extracellular protein was measured by the Bio-Rad Protein Assay Kit (Bio-Rad, USA) following the manufacturer's instructions.

For sporulation assay, an aliquot of 10 μL (10^8^/L) spores of the *T. reesei* strains were cultivated on MEA plates at 28°C for 7 days to obtain mature conidia, which were collected and counted using a hemocytometer.

### Construction of *T. reesei* Mutants Overexpressing and Deleting Various Putative Regulatory Genes

All primers used for strain construction and confirmation were listed in Table S1. In order to study the effect of AZFP-U5 on cellulase synthesis in *T. reesei*, its nucleic acid sequence was amplified by PCR with the primers pCB303-U5-F and pCB303-U5-R, and the PCR product was ligated into the expression vector pCB303, then transformed into *T. reesei* Rut-C30 using the ATMT method. After confirmation of the correct transformants, the cellulase activities of at least three randomly selected transformants were determined.

For construction of the *T. reesei* mutants overexpressing endogenous putative regulators, the PCR products of 15 corresponding putative regulatory genes amplified from genomic cDNA were fused, respectively, into the pCZF3 vector (Zhang et al., 2018a) digested by *Nco* I and *Xba* I using Seamless Cloning Master Mix (Sangon Biotech, China). Each ligated product was then transformed into *T. reesei* Rut-C30 using the ATMT method, putative regulatory genes were overexpressed individually, driven by the pyruvate decarboxylase (*pdc*) promoter.

Regarding the construction of *TrC30_93861* (*ypr1*) or *TrC30_74374* knockout strains, the 2,000-bp upstream and downstream region of the corresponding genes were amplified from *T. reesei* Rut-C30 genome as the homologous arms individually. The hygromycin B resistance gene *hph* was employed as the selected marker, and the expression cassette was amplified from the pCZF3 vector using primers hphCAS-F and hphCAS-R. Then, the *hph* expression cassette together with the 2,000-bp flanking arms were cloned into plasmid pZF-MCS at *Hin*d III and *Eco*R I sites using ClonExpress™ II One Step Cloning Kit (Vazyme, China), resulting in the plasmid pZF-93861D and pZF-74374D. The plasmids pZF-93861D and pZF-74374D were then introduced into *T. reesei* Rut-C30 Δ*ku70* by the ATMT method. The correct deletion mutants were verified using four pairs of specific deletion primers, and the design scheme for strain constructions and verifications are provided in Figure S3.

### Determination of the Copy Number and Integrated Site of AZFP-U5

To determine the copy number of the AZFP encoding gene *AZFP-U5* in the *T. reesei* U5, genomic DNA of *T. reesei* U5 was isolated and used as the template for quantitative PCR (qPCR) with the primers AZFPU5-F and AZFPU5-R. The qPCR method is the same as that described by Solomon et al. (2008), in which *tef1*α (translation elongation factor 1-alpha) was used as a control for a single copy. Correspondingly, the integrated site of the *AZFP-U5* was determined by the thermal asymmetric interlaced PCR (TAIL-PCR) according to the procedure described elsewhere (Liu and Chen, 2007). Additionally, the copy number of *hph* expression cassette in respective regulator deletion mutants was verified by qPCR with the primers hphqPCR-F and hphqPCR-R. The primers used for qPCR and TAIL-PCR are listed in Table S2.

### Comparative Transcriptome Analysis

*T. reesei* U5 and Rut-C30 were grown in the MA medium with cellulose as the carbon source, then, the total RNAs from the mycelia were isolated at 24 and 48 h using the RNA extraction kit (Sangon Biotech, China). RNA sequencing (RNA-Seq) was performed at the BGI Company (Shenzhen, China) using the Illumina HiSeq2000 system. Gene expression levels were calculated using the fragments per kilobase of exon model per million reads (FPKM) method (Mortazavi et al., 2008). Threshold values of |log_2_ FC (fold change of U5 to Rut-C30)| ≥1 and FDR (False Discovery Rate) <0.001 were used to judge the significance. Genes were annotated according to the *T. reesei* Rut-C30 genome annotation, and proteins involved in CAZymes, transcription factors, transporters, secondary metabolism clusters, protein processing, and secretion were referred to genome database of Joint Genome Institute (https://mycocosm.jgi.doe.gov/pages/search-for-genes.jsf?organism=TrireRUTC30_1) (Grigoriev et al., 2014).

To elucidate the effects of *ypr1* in pigment production, *T. reesei* Δ*ypr1* and its parent strain *T. reesei* Rut-C30 Δ*ku70* were cultured in the MA medium with 2% glucose as a carbon source, then the mycelia of these two strains at 24 h were collected for total RNA extraction. The process of transcriptome analysis for *T. reesei* Δ*ypr1* and Rut-C30 Δ*ku70* is the same as described above for *T. reesei* U5 and Rut-C30. The mRNA profile for specific genes was also analyzed for verification of RNA-seq results with corresponding primers (Table S1). The NCBI accession numbers for transcriptome data of *T. reesei* U5 and Rut-C30, *T. reesei* Δ*ypr1* and Rut-C30 Δ*ku70* are PRJNA612708 and PRJNA613769, respectively.

### Enzymatic Hydrolysis of the Pretreated Biomass

Biomass hydrolysis efficiency of cellulase produced by *T. reesei* was measured using alkaline pretreated corn stover (APCS). Corn stover was pretreated by 2% (w/v) sodium hydroxide and 2%(w/v) hydrogen peroxide, washed to reach neutral pH, and lignocellulose components and hydrolysis were measured according to the method described previously (Zhang et al., 2016). Hydrolysis experiment was carried out at 5% total solids loading with APCS as the substrate, and the cellulase loading was 30 mg per gram of dry biomass. The hydrolysis conditions are incubation at 50°C, pH 4.8, shaking at 150 rpm for 48 h, and samples were collected at an interval of 12 h. The sugar ingredients and concentrations released in the hydrolysate were analyzed by HPLC. The experiments were performed in triplicate, and the results are represented as the average values ± standard deviation (SD).

## Results

### Cellulase Properties of *T. reesei* Rut-C30 and U5

The mutant strain *T. reesei* U5 with improved cellulase production was selected from the *T. reesei* Rut-C30 transformants transformed by the AZFP library (Zhang et al., 2016). The AZFP sequence from the transformant *T. reesei* U5 was named as AZFP-U5, and the corresponding gene *AZFP-U5* was found to integrate into the genome as a single copy. The single integration site was identified to be located at the scaffold 33: 124903–124921 of *T. reesei* Rut-C30 chromosome, which is the intergenic region between terminators of two genes of *TrC30_91501* and *TrC30_91570*, indicating that AZFP-U5 functions without disrupting other functional genes.

**Figure 1:**
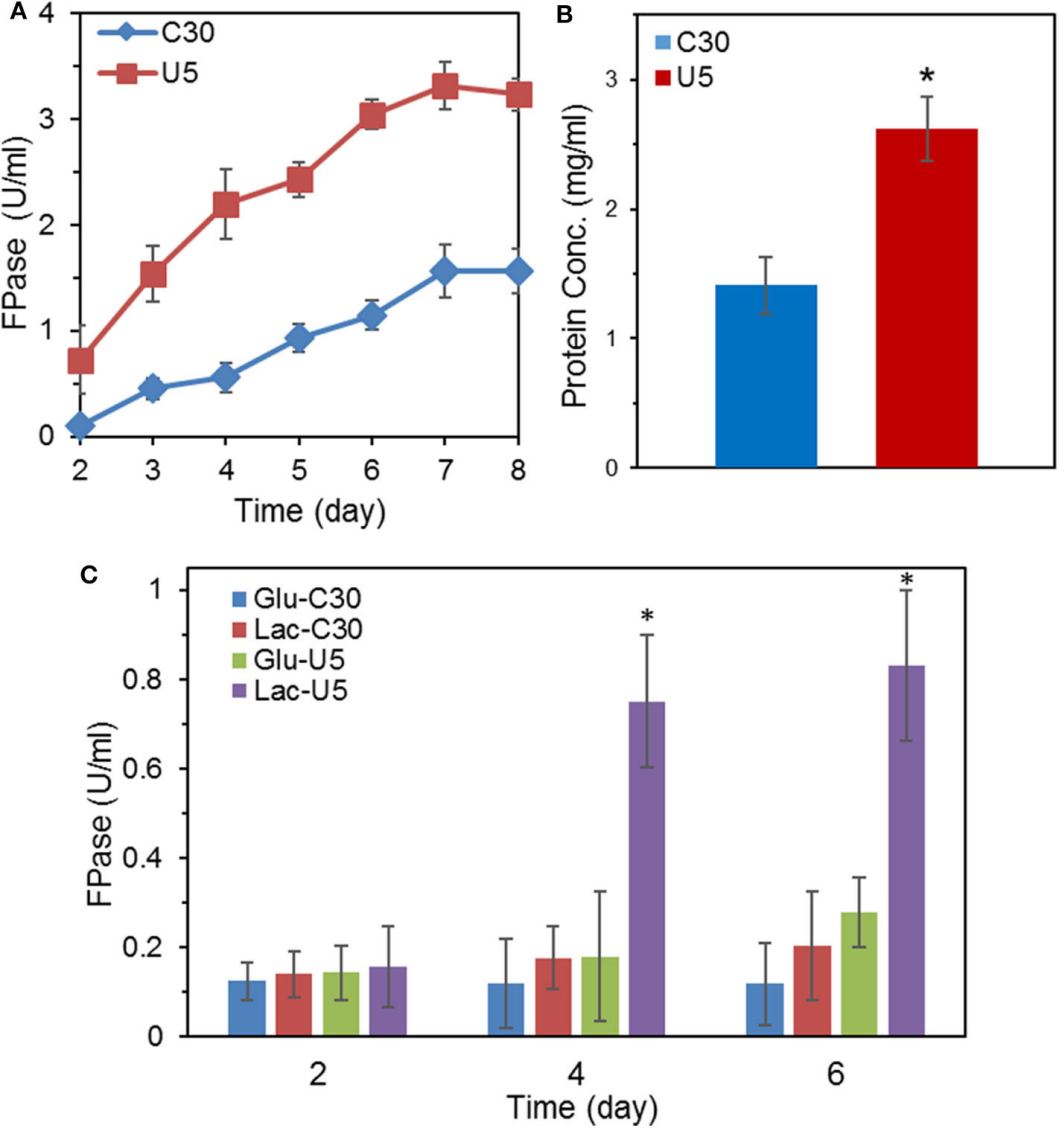
Comparison of FPase activities and secreted protein concentration between the mutant *T. reesei* U5 and the parental strain Rut-C30. **(A)** FPase activities of *T. reesei* U5 and Rut-C30 in MA medium with 2% cellulose and 2% wheat bran as carbon source. **(B)** Extracellular protein concentration of *T. reesei* U5 and Rut-C30 at 7th day's fermentation. **(C)** FPase activities of *T. reesei* U5 and Rut-C30 in MA medium with 2% glucose or lactose as a carbon source. Asterisks indicate significant differences from reference strains (*p < 0.05, Student's *t*-test).

With cellulose as the carbon source in the MA medium, the FPase activity of *T. reesei* U5 was 2.1-folds of that from *T. reesei* Rut-C30, reaching 3.31 U/mL on the 7th day, while the FPase activity in the parental strain was 1.56 U/mL (Figure 1A). In addition to increasing the total FPase activity, U5 cellulase also exhibited superior profile for various cellulase components. As shown in Figure S1, the endoglucanase activity of *T. reesei* U5 reached 17.23 U/mL, increased by 1.88 times to that from Rut-C30. The activity of β-glucosidase secreted by U5 strain was 2.76 U/mL, much higher than that from *T. reesei* Rut-C30 (1.09 U/mL). The extracellular xylanase activities of *T. reesei* U5 and Rut-C30 were 596 and 388 U/mL, respectively. Meanwhile, the extracellular proteins of those two strains were significantly different, the amount of protein secretion from *T. reesei* U5 was increased by 86% relative to that from Rut-C30. The extracellular protein concentration from *T. reesei* U5 reached 2.62 mg/mL, while it was about 1.41 mg/mL for Rut-C30 (Figure 1B). When the re-constructed *AZFP-U5* plasmid was transformed into *T. reesei* Rut-C30, the enhanced cellulase production phenotypes were observed in all three randomly selected mutants.

In addition to cellulose, lactose also has an inducing effect on cellulase production. The FPase activities between cellulase U5 and Rut-C30 were found to be significantly different in the fermentation medium containing 2% glucose or lactose as carbon sources, as shown in Figure 1C. On the 6th day of fermentation in glucose medium, the FPase activity from U5 cellulase reached 0.28 U/mL, much higher than that in *T. reesei* Rut-C30 (0.12 U/mL). The FPase activities of cellulase U5 and Rut-C30 in the lactose medium were 0.83 and 0.20 U/ml, respectively, and over 4 times improvement was observed in cellulase U5 compared to that from Rut-C30, which demonstrates that *T. reesei* U5 partially abolished the CCR effect in the presence of glucose and lactose.

**Figure 2:**
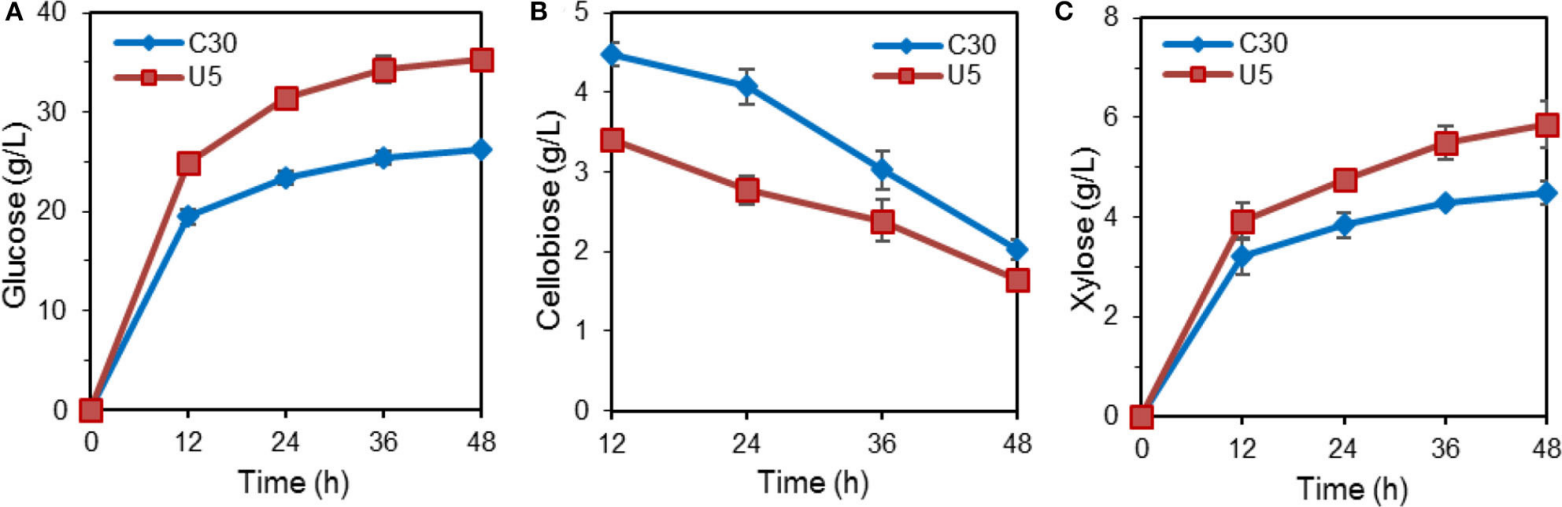
Hydrolysis of pretreated corn stalk by cellulase produced by *T. reesei* U5 and Rut-C30. **(A)** Glucose releasing concentration. **(B)** Cellobiose releasing concentration. **(C)** Xylose releasing concentration.

The hydrolytic ability for pretreated lignocellulose was further evaluated. In the course of hydrolysis on alkali pretreated corn stover by crude cellulase from *T. reesei* Rut-C30 and U5, the released glucose reached 35.22 g/L (corresponding to ~95% cellulose conversion) by the crude cellulase from *T. reesei* U5, which increased by 33.9% compared to that from Rut-C30 (26.30 g/L) at 48 h. Meanwhile, 31% higher xylose yields were also observed by the cellulase produced by *T. reesei* U5. At 48 h, slightly lower residual cellobiose was observed using the cellulase U5 (1.65 vs. 2.03 g/L from the parental strain) (Figure 2).

### Transcriptional Changes of Major Cellulases and Transcription Factors

**Figure 3:**
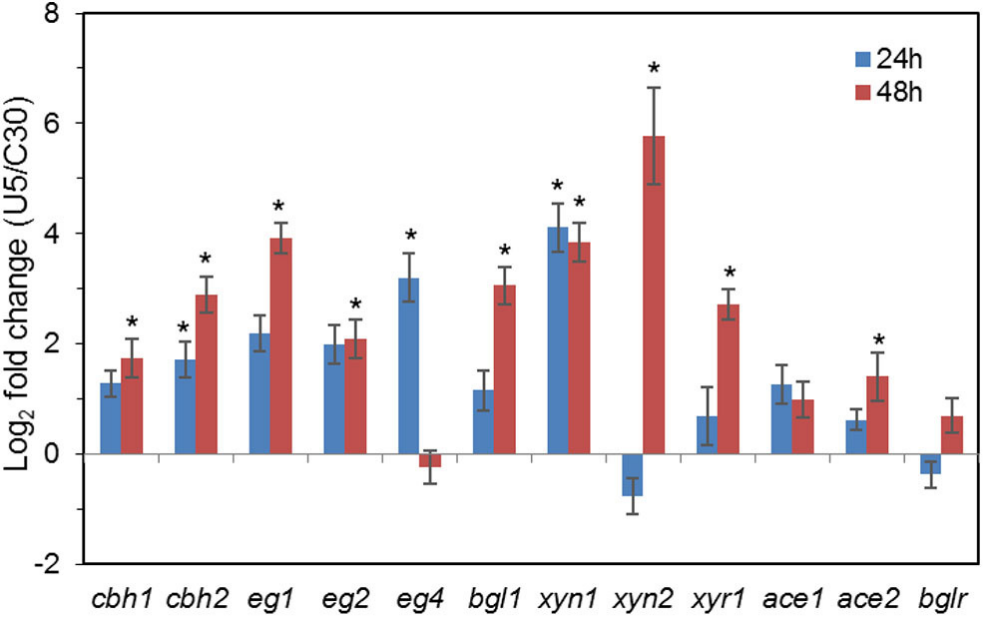
Transcription of genes encoding major cellulases and transcription factors in *T. reesei* U5 and Rut-C30. Asterisks indicate significant differences from reference strains (*p < 0.05, Student's *t*-test).

As shown in Figure 3, transcription levels of the five major cellulase components in *T. reesei* U5, including the major cellulase genes *cbh1/2, eg1/2*, and *bgl1*, increased significantly (2–16-fold) compared with that of *T. reesei* Rut-C30. In the U5 strain, the transcript of *bgl1* gene increased over 1-fold, and transcription of the endoglucanse genes *eg1* and *eg2* increased by 3-fold at 24 h compared to that in *T. reesei* Rut-C30. At 48 h, the transcription levels of main cellulase genes in *T. reesei* U5 increased by 4–16 times compared to that from Rut-C30, and the transcription levels of the two major xylanase genes *xyn1* and *xyn2* improved 32 and 128 times, respectively. Meanwhile, transcription of the major transcription factor *xyr1* gene also increased, of which the transcript in *T. reesei* U5 was 5-fold to that in Rut-C30 at 48 h. The transcription levels of other regulators for cellulase regulation were also remarkably increased, which include *ace1, ace2*, and *bglr*.

The artificial regulator AZFP-U5 (Table S3) is composed of C_2_H_2_ type zinc finger and Gal4 effector domain. The zinc finger sequence of AZFP-U5 is VSSR-RDER-ISNR-QNTQ, and the potential binding site is GYDGHGGAHATA (where Y represents C/T, D represents G/A/T, and H represents C/T/A) (Park et al., 2003). After querying the corresponding binding sites in the Rut-C30 genome, 47 from 106 potential binding sites were identified to be located within 1,500 bp upstream of the coding region of specific genes. These 47 genes might be direct targets of the artificial regulator AZFP-U5, of which the profiles of transcripts and potential functions were provided in Additional file 1. These genes are related to carbohydrate and nucleic acid metabolism, transcriptional regulatory signaling pathways (transcription factors, protein kinases), protein folding and secretion, putative extracellular proteins, 13 of these 47 potential genes are classified as proteins with unknown functions. Since the transcription of major cellulase and hemicellulase genes in *T. reesei* U5 has increased significantly, it is speculated that proteins related to signal transduction and transcription regulation play a key role for AZFP-U5's regulatory effects. The putative sugar transporter gene (*TrC30_85306*), putative histidine kinase (*TrC30_129764*), and putative protein kinase (*TrC30_93459*) may participate in the cellulase induction and gene expression. The effect of *TrC30_129764* on cellulase production was further tested together with the other regulators, which were described in the section Exploration of New Regulators for *T. reesei* Cellulase Production.

### Comparative Transcriptome Analysis of *T. reesei* U5 and Rut-C30

The total transcriptome reads of *T. reesei* U5 and C30 at 24 h were 48,803,620 and 49,017,490, respectively. At 48 h, the reads were 48,988,334 and 48,998,278, respectively, which have met the coverage assessment for the randomness and sequencing saturation assessment. Differentially expressed genes (DEGs) showed that there were 2,203 up-regulated genes and 786 down-regulated genes at 24 h, and 1,500 up-regulated genes and 383 down-regulated genes were observed at 48 h.

In addition to the five glycoside hydrolase genes, five down-regulated genes were randomly selected for RT-qPCR to verify the reliability of the transcriptome data (Figure S2). Similar trends of difference were observed by the RT-qPCR analysis and the transcriptome analysis. Therefore, the transcriptome data of *T. reesei* U5 and C30 can be considered reliable for further analysis.

#### Gene Ontology (GO) and Functional Category of DEGs

**Figure 4:**
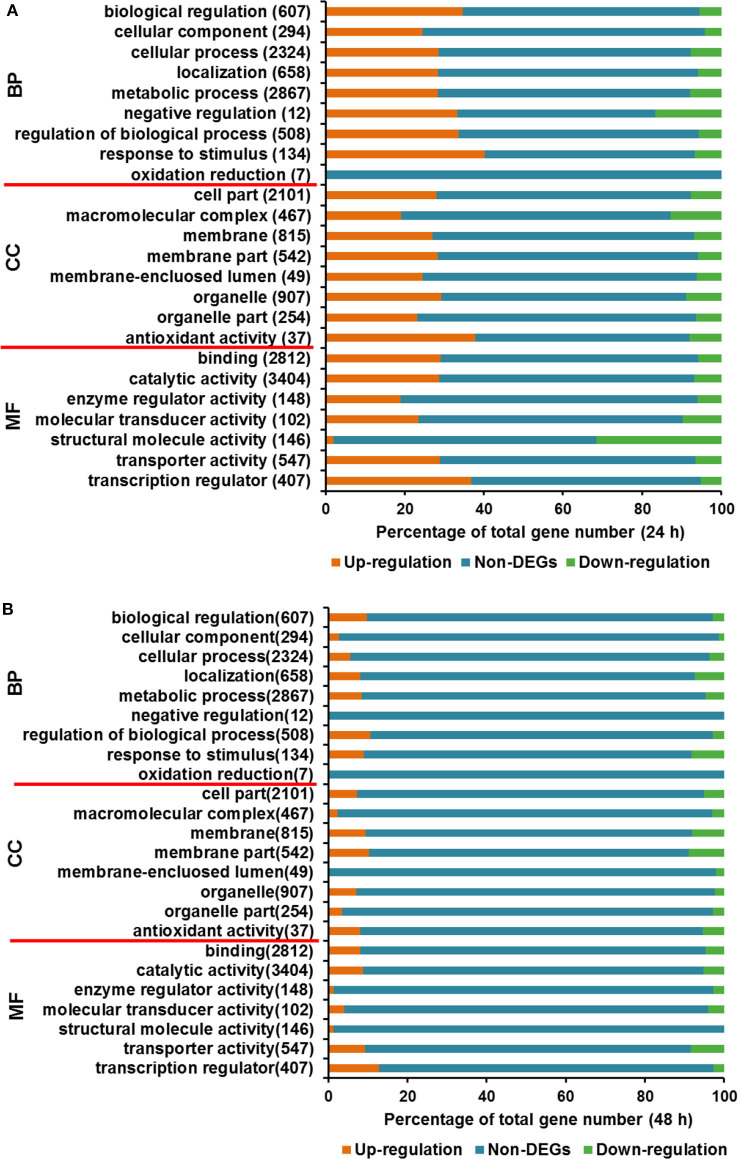
Gene Ontology analysis for DEGs between *T. reesei* U5 and Rut-C30. **(A)** Gene Ontology analysis at 24 h. **(B)** Gene Ontology analysis at 48 h. BP, biological process; CC, cellular component; MF, molecular function.

In the course of transcriptome analysis, some functional DEGs exceed 40% of the total corresponding functionally classified genes in several GO terms at 24 h, including biological regulation, cell process regulation, stress response, antioxidant activity, transport activity, and transcriptional regulation (Figure 4A). At 48 h, the functional DEGs accounting for over 15% of the overall functional classification genes are located in the following categories, localization function, stress response, membrane fraction, transport activity, and transcription factors (Figure 4B). GO analysis at two time points indicated that the transcriptional changes of functional DEGs, such as stress response, transporter and transcription factor proteins, were relatively significant.

**Figure 5:**
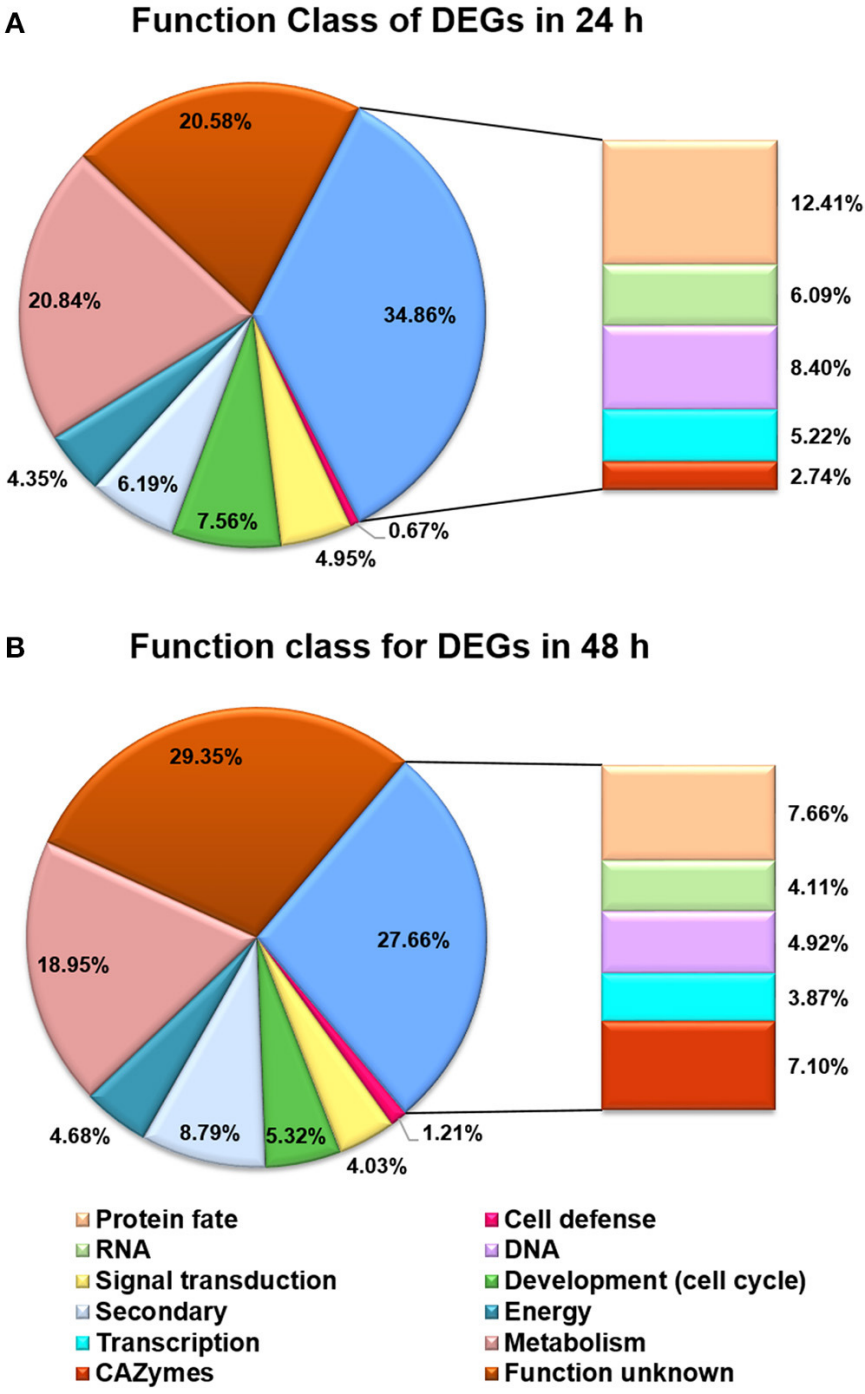
Functional categories for DEGs between *T. reesei* U5 and Rut-C30. **(A)** Functional categories for DEGs at 24 h. **(B)** Functional categories for DEGs at 48 h. Protein fate (protein translation, folding, modification, sorting, and secretion); RNA (RNA synthesis, processing, modification, surveillance and degradation); DNA (DNA replication, precursor, repairs).

Besides the GO analysis, functional category of DEGs between *T. reesei* U5 and Rut-C30 was performed to evaluate statistically significant enrichment of gene functions (Figure 5). A number of DEGs were associated with metabolic functions, which account for 20.84 and 18.95% of total DEGs at 24 and 48 h, respectively. More importantly, the functional groups of DEGs included in protein fate (protein translation, folding, modification, sorting, and secretion), RNA processing (synthesis, modification, surveillance, and degradation), and transcription and signal transduction, take up 12.41%, 6.09%, and 10.17% of the total DEGs at 24 h, and 7.66%, 4.11%, and 7.90% at 48 h, respectively (Table S4, Additional file 2). The functional classification of DEGs demonstrates that RNA and protein processing kept active in *T. reesei* U5, especially at 24 h, which were analyzed in detail below.

#### Analysis of Genes Encoding Transcription Factors, GHs and Major Transporters

**Figure 6:**
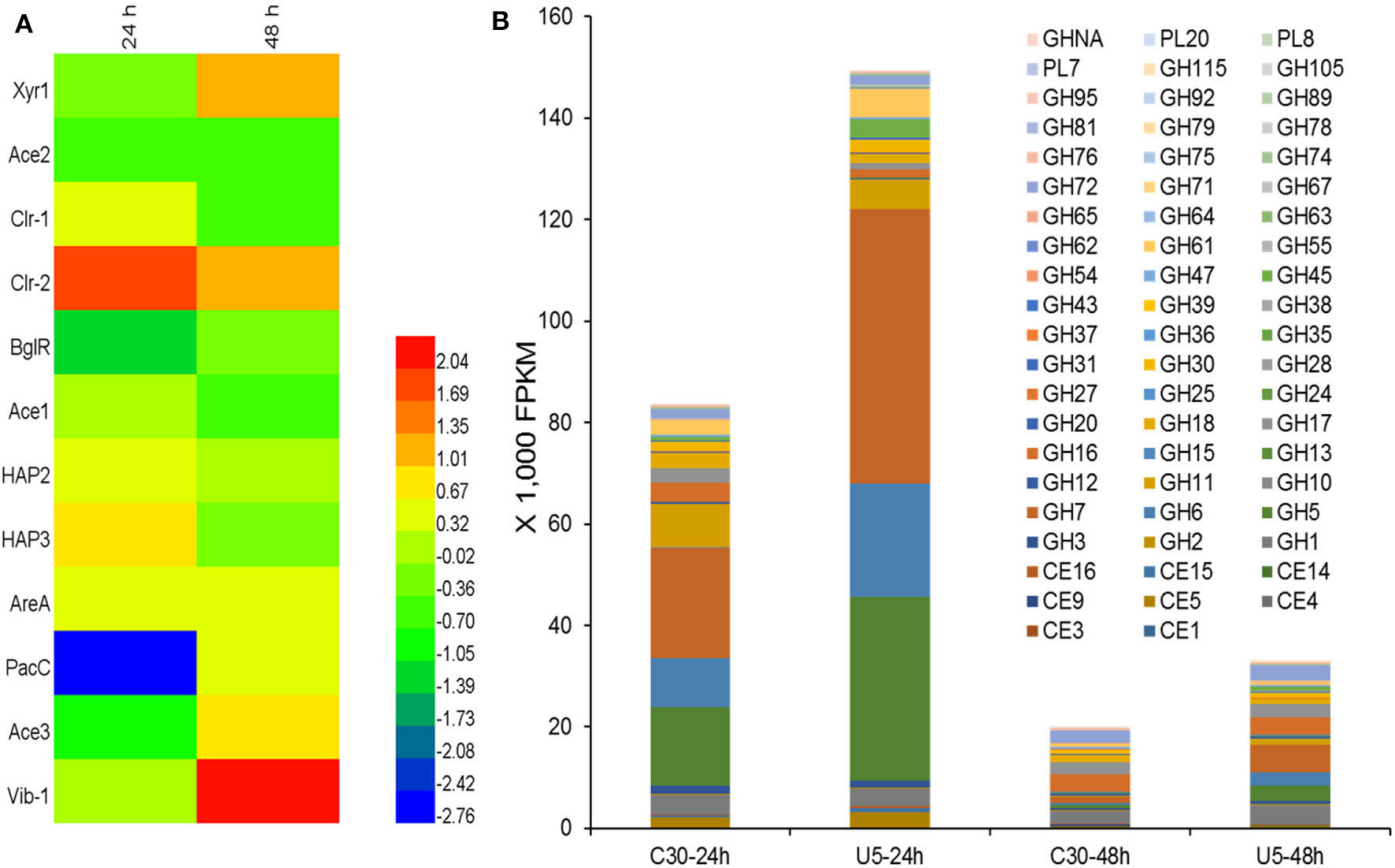
Differential transcription of genes encoding potential transcription regulators **(A)** and CAZymes **(B)**. CE, Carbohydrate esterase; GH, Glycoside hydrolase; GHNA, Glycoside hydrolase in uncategorized family; PL, Polysaccharide lyase.

Regulators that potentially play crucial roles in the induction and production of cellulase in *T. reesei* were analyzed. Totally 420 genes in the *T. reesei* genome were predicted to encode transcriptional regulators, from which the transcripts of 223 genes (195 up-regulated; 38 down-regulated) and 91 genes (68 up-regulated; 23 down-regulated) changed significantly at 24 and 48 h, respectively (Additional file 3). Although the transcription levels of main cellulases improved remarkably in *T. reesei* U5 during the course of analysis, the known major transcription factor genes, such as *xyr1, ace2, ace3*, and *vib1*, have not changed consistently at 24 h, whose corresponding transcripts increased to a certain extent at 48 h in *T. reesei* U5 (Figure 6A). At 48 h, the transcription of *xyr1* gene in *T. reesei* U5 improved more than 2-folds compared to that in Rut-C30. Meanwhile, the transcript level of the positive regulatory gene *vib-1* in *T. reesei* U5 reached 4-fold compared to that in Rut-C30 at 48 h. The Clr-2 homologous protein is the important glycoside hydrolase positive regulator in *Penicillium oxalicum* and *Neurospora crassa* (Coradetti et al., 2012; Li et al., 2015), whose transcript levels in *T. reesei* U5 were 3 and 2-folds to that of Rut-C30 at 24 and 48 h, respectively. Protein functions of several differentially expressed transcription factors have been identified, among which overexpression of the gene *TrC30_81999* has a positive regulatory effect on cellulase production of *T. reesei* (Kubicek, 2013), and the gene *TrC30_101064* encoding the transcription factor Xpp1, has a feedback inhibitory effect on xylanase induction under high-concentration xylose induction conditions (Derntl et al., 2015). The transcripts of *TrC30_74374* and *TrC30_96554* related to light-mediated regulation in *T. reesei* U5 were up-regulated 16-fold and down-regulated 8-fold at 24 h (Tisch and Schmoll, 2013). The results above provide a basis for further exploring novel regulators for cellulase synthesis and regulation. Interestingly, the transcripts of repressor genes for cellulase production, *ctf1* (*TrC30_10530*) (Meng et al., 2020) and *rce1* (*TrC30_6520*) (Cao et al., 2017), always improved about 1-2-fold in *T. reesei* U5 in the time course compared to that in Rut-C30. The velvet family proteins Ve1 and Vel2 possess positive effect on cellulase, however, the transcript levels of *ve1* (*TrC30_98965*), *vel2* (*TrC30_102739*) in *T. reesei* U5 were similar as that in Rut-C30. Genes *gcn5* (*TrC30_82854*) and *lae1* (*TrC30_9778*) encoding histone acetyltransferase and methyltransferase, respectively, whose functions were verified to be relevant to cellulase regulation (Seiboth et al., 2012; Xin et al., 2013; Liu et al., 2016), also showed no significant changes.

The main transcription factors Xyr1 and Cre1 proteins could affect transporter family proteins in *T. reesei*, especially major facilitator superfamily (MFS) (dos Santos Castro et al., 2014, 2016), of which several sugar transporters transmit extracellular carbon-induced signals or transport oligosaccharide inducer into cell, such as the sugar transporters Cdt1 and Cdt2 in *N. crassa* (Znameroski et al., 2013), CltB in *Aspergillus nidulans* (dos Reis et al., 2016), and Crt1 (Zhang et al., 2013) in *T. reesei*. About 448 genes in the *T. reesei* genome are predicted to have transporter function (Chaudhary et al., 2016), among which 51 transporter genes changed significantly (|Log_2_FC|>1, *p* < 0.05). However, the transcription of *crt1* (*TrC30_109243*) kept the same level between *T. reesei* U5 and *T. reesei* Rut-C30. Besides ATP-binding cassette transporter superfamily genes, the transcripts of several predicted sugar transporters also showed the remarkable changes, in which the transcriptional levels of *TrC30_7623, TrC30_95062, TrC30_97401*, and *TrC30_12083* gene were up-regulated at least 2-fold, whereas transcripts of *TrC30_38765, TrC30_136619, TrC30_124396*, and *TrC30_133544* decreased at least 1-fold in comparison to that of Rut-C30. The sugar transporters above may play crucial roles on inducer transportation or transmission of carbon signals in *T. reesei* U5, which may re-modulate the profile of cellulase induction and production.

#### Transcription Analysis of Extracellular Protein Processing

As shown in Figure 6B, the transcript quantities of the major cellulases belonging to GH5, GH6, and GH7 families accounted for 56.2% of the total transcription of glycoside hydrolases (82,000 FPKM) in cellulase Rut-C30 at 24 h. However, the corresponding ratio is up to 75.5% for cellulase U5, whose total transcripts is 148,000 FPKM. The total transcripts of extracellular glycoside hydrolases, especially, major induced cellulases genes, such as genes *cbh1, cbh2, eg1*, and *eg2*, decreased significantly at 48 h compared to that at 24 h in two *Trichoderma* strains, probably due to repression under secretion stress (RESS) (Pakula et al., 2003). At 48 h, the transcription of extracellular major glycoside hydrolase genes in the two strains decreased significantly compared to that at 24 h, and the total FPKM values of extracellular protein U5 and Rut-C30 at 48 h decreased to 22–25% of that at 24 h, meanwhile, the transcripts of genes encoding the major cellulases from GH5, GH6, and GH7 families occupied 11.2% of the total cellulase transcripts in cellulase Rut-C30, 33.6% in cellulase U5 (Additional file 4). Furthermore, the transcripts of three major proteases genes (*TrC30_111063, TrC30_122545*, and *TrC30_26076*) in *T. reesei* U5 kept the same level to that of Rut-C30, which may exert negative effect on the stability of extracellular glycoside hydrolases (Qian et al., 2019).

Genes related to protein processing, folding, transporting, and secretion exhibited significant changes in *T. reesei* U5 compared to that of Rut-C30. At 24 h, the transcription levels of 14 aminoacyl-tRNA synthetase genes in *T. reesei* U5 were significantly higher than that in Rut-C30, whose transcript levels increased by 2 to 5.6 times. The transcriptional levels of genes encoding endoplasmic reticulum-related proteins responsible for protein processing and folding were also changed in *T. reesei* U5, over 40% endoplasmic reticulum protein, transporting and sorting proteins were significantly up-regulated in *T. reesei* U5, which may be beneficial to protein secretion (Additional file 5). The transcription levels of genes related to N-glycosylation modification in the endoplasmic reticulum were significantly increased (Ruddock and Molinari, 2006). In addition, the transcription levels of genes such as mannosyl oligosaccharide glucosidase (*ERManI* homolog), α-1, 3 glucanase (*GlcII* homolog), and protein oligosaccharyltransferase (*UGGT* homolog) increased by 2–3.5 times, which all are the key glycosylation enzymes and hydrolases in the endoplasmic reticulum for protein processing. Furthermore, the *EDEM* homolog gene encoding the marker protein of the endoplasmic reticulum associated degradation pathway, was significantly up-regulated (8-fold) compared to that in Rut-C30 (Wang and Hebert, 2003). The transcription of the eukaryotic translation initiation factor 2α kinase (*PERK* homolog) gene was improved 1.3-fold, which is a characteristic enzyme for endoplasmic reticulum stress response (Harding et al., 1999). Results above indicate the enhanced effect of endoplasmic reticulum-associated degradation (ERAD) on protein processing for glycoside hydrolases in *T. reesei* U5 at 24 h. However, at 24 h, the transcription levels of the genes encoding disulphide isomerase Pdi and ER-resident transmembrane kinase Ire1 proteins were not up-regulated, the transcript levels of *hac1* gene encoding regulator for UPR, and *bip1* gene encoding the luminal binding protein, an ER-localized member of the HSP70 family, also kept in similar level between *T. reesei* U5 and Rut-C30 at 24 h, which suggests that the unfolded protein response (UPR) was not changed in *T. reesei* U5. The positive function of genes above for protein processing in endoplasmic reticulum in *T. reesei* have been confirmed before (Saloheimo et al., 2003; Gao et al., 2018).

The ERAD and misfolding protein degradation pathway are always coupled. At 24 h, transcription quantity of the ubiquitination-proteasome system-related genes in *T. reesei* U5 was significantly up-regulated, which is important to reduce the pressure on the endoplasmic reticulum owing to the accumulation of post-translated glycoside hydrolases. The transcription of genes encoding Derlin and Ubx family proteins involved in the ERAD pathway was increased in *T. reesei* U5, facilitating the entry of misfolding protein into the proteasome. In the course of protein ubiquitination, genes coding the ubiquitin-activating enzyme E1 family, the ubiquitin-binding enzyme E2 family, and the ubiquitin ligase E3 family in *T.reesei* U5 were all increased to various degrees, among which genes encoding ubiquitin-activating enzyme UBE1, UBLE1A, UBLE1B, and UBE1C were up-regulated by over 3-fold, and the ubiquitin-binding enzyme UBE2Q gene was up-regulated by 6-fold. Correspondingly, transcription levels of the proteasome subunit genes in *T. reesei* U5 also improved to various degrees, at least twice as high as that in Rut-C30 at both time points. Transcripts of genes in the ERAD pathway, as well as ubiquitination and proteasome-related genes increased remarkably, indicating that the protein processing of *T. reesei* U5 has begun speedily at 24 h, which resulted in a large increase in extracellular cellulase protein secretion in *T. reesei* U5 compared to that in *T. reesei* Rut-C30.

In addition, there are several significantly different transcriptional levels in metabolic pathways or cellular physiological activities between *T. reesei* U5 and Rut-C30 at 24 h, and the transcript level of genes in the following groups always improved remarkably in *T. reesei* U5: RNA polymerase II subunit and its basic transcriptional regulatory factors, mRNA regulatory pathways, RNA degradation, peroxisomes, CoA synthesis pathways, N-glycoside synthesis pathways, ammonia Acyl-tRNA synthesis pathway, nicotinamide metabolism pathway, fatty acid metabolism pathway, endoplasmic reticulum membrane protein processing, and proteasome complex (Additional file 2).

### Exploration of New Regulators for *T. reesei* Cellulase Production

In the transcriptome data analysis of *T. reesei* U5, putative regulatory genes changing significantly at both 24 and 48 h are likely to be targets for activating the transcription of cellulase genes. Therefore, 15 genes possessing predicted regulatory function were selected as targets for further analysis. The predicted protein-encoding genes screened are shown in Table 1, which mainly include the fungal Zn_2_Cys_6_ type binuclear cluster transcription factor, histone kinase, LysR family regulatory proteins, helix-transition-helix regulatory proteins, and NmrA family regulatory proteins.

**Table 1:** Predicting differentially expressed genes with regulatory functions by overexpression of AZFP-U5.

**Model ID**	**Prot ID**	**Log_**2**_FC (24 h)**	**Log_**2**_FC (48 h)**	**Annotation**
estExt_Genemark1.C_1_t10198	23164	1.80	0.66	Fungal Zn(2)-Cys(6) binuclear regulator
estExt_Genemark1.C_1_t20246	23425	2.20	0.59	Fungal Zn(2)-Cys(6) binuclear regulator
estExt_fgenesh1_pg.C_110229	141745	0.68	1.72	Fungal Zn(2)-Cys(6) binuclear regulator
estExt_Genewise1Plus.C_17_t20040	102019	2.50	0.62	Fungal specific transcription factor
fgenesh1_pm.8_#_7	129764	2.14	0.82	Signal transduction histidine kinase
fgenesh1_pg.24_#_1	38417	0.75	6.17	LysR family regulatory protein
estExt_fgenesh2_kg.C_250078	124902	8.92	0.62	Helix-turn-helix containing protein
e_gw1.12.485.1	81999	6.73	0.73	Fungal Zn(2)-Cys(6) binuclear regulator
fgenesh1_pg.24_#_106	38522	5.72	1.49	Fungal specific transcription factor
estExt_Genewise1Plus.C_10_t10436	99728	3.22	1.93	Fungal specific transcription factor
e_gw1.47.20.1	93160	11.76	2.52	Fungal Zn(2)-Cys(6) binuclear regulator
estExt_Genemark1.C_270039	26551	5.37	1.96	NmrA-like family regulator
e_gw1.3.267.1	72675	4.98	1.36	Fungal Zn(2)-Cys(6) binuclear regulator
estExt_Genewise1Plus.C_1_t10373	93861	4.45	1.05	Fungal Zn(2)-Cys(6) binuclear regulator
e_gw1.4.341.1	74374	3.96	1.32	Fungal Zn(2)-Cys(6) binuclear regulator

**Figure 7:**
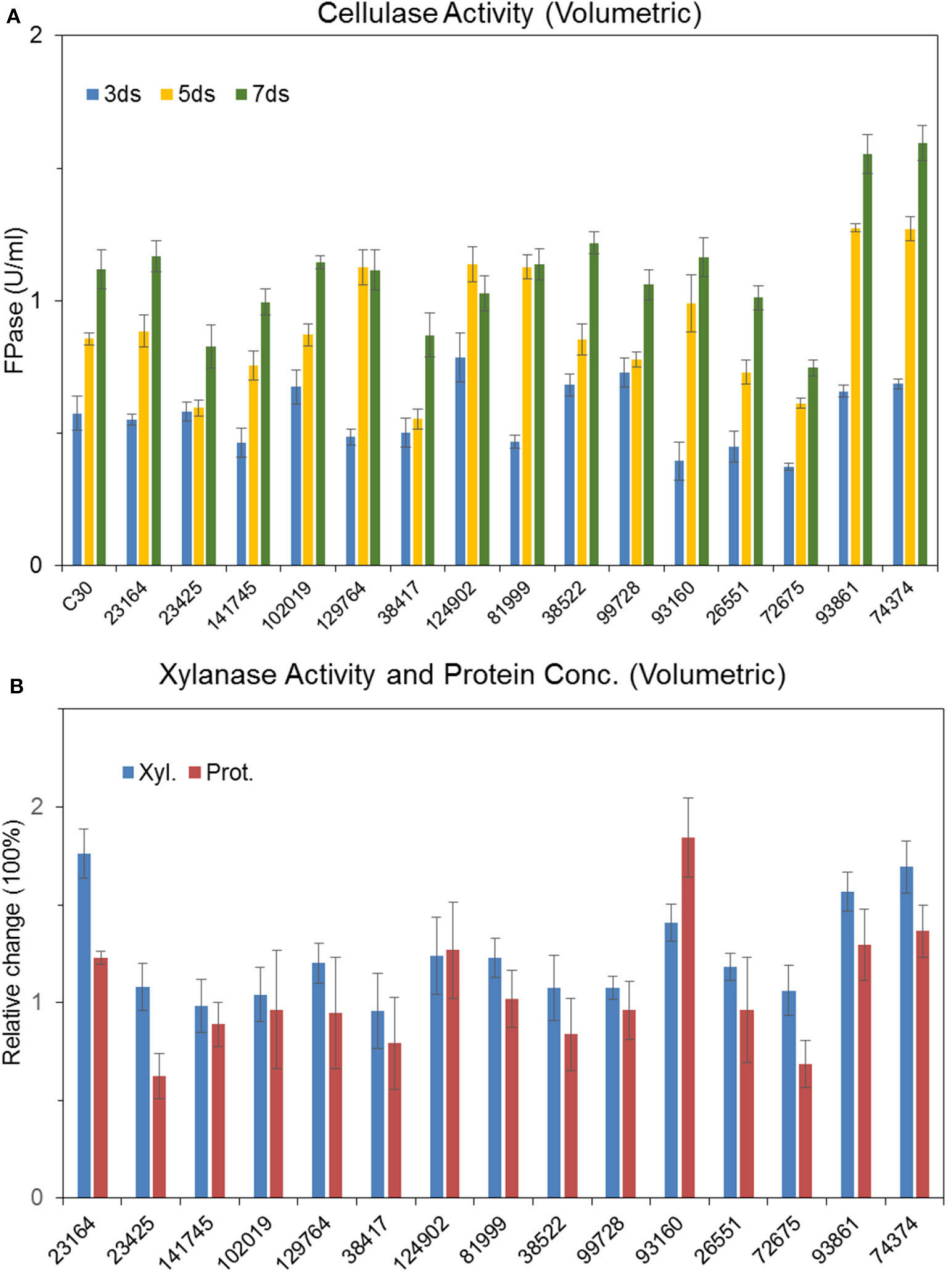
Effects of overexpression of 15 transcription factors on cellulase production Cellulase **(A)**, xylanase and protein productions **(B)** by *T. reesei* Rut-C30 recombinant strains overexpressing the 15 candidate transcription factors.

Recombinant strains with overexpression of the 15 putative regulators were obtained. With the exception of *T. reesei* Tr93861 strain overexpressing gene *TrC30_93861*, whose spore-forming ability was reduced significantly and mycelium growth was impaired, the other strains did not have significant changes in growth and mycelial morphology compared to *T. reesei* Rut-C30. Notably, the TrC30_93861 protein homolog in the wild-type strain *T. reesei* QM6a has been identified as the transcription factor Ypr1 (Derntl et al., 2016), so *TrC30_93861* gene is named as *ypr1* in the following text. Through the fermentation performance of the transformants overexpressing 15 putative regulatory genes, it can be deduced that these regulators, when individually overexpressed, did not exert any positive regulation on cellulase production, and even the overall enzyme activities from some certain transformants showed a decreased level compared to Rut-C30, as shown in Figure 7. Gene *TrC30_129764* is the potential target gene regulated by AZFP-U5 directly, whose transcript also improved significantly in the *T. reesei* U5. However, *TrC30_129764* overexpressed strain showed similar cellulase activity profile with that in Rut-C30, which suggest that TrC30_129764 is not a crucial factor for cellulase regulation. Furthermore, the transcript level of gene *TrC30_38522* encoding a putative transcriptional regulator in *T. reesei* U5 improved by over 50 and 2-fold at 24 and 48 h compared to that in Rut-C30, whose expressional level also elevated several folds when *T. reesei* Rut-C30 was cultured into the sugarcane bagasse medium (Borin et al., 2018). However, no difference was observed for cellulase production when *TrC30_38522* was overexpressed in *T. reesei* Rut-C30. Among the transformants, the strains overexpressing genes *ypr1* and *TrC30_74374* showed 38.8% and 42.7% higher FPase activities than that of Rut-C30, respectively. Meanwhile, the corresponding extracellular protein secretion increased by 29.44% and 36.54%, respectively.

### Possible Crosslink Between Cellulase Production and Pigment Secretion

In order to further study the functions of *ypr1* or *TrC30_74374* in *T. reesei* Rut-C30, *ypr1*, and *TrC30_74374* gene knockout strains Δ*ypr1* and Δ*74374* were constructed and verified individually with the correct integration as a single copy (Figure S4). Unexpectedly, no change in cellulase activities were observed in strains Δ*ypr1* and Δ*74374* as to that in the control strain *T. reesei* Rut-C30 (Figure S5), suggesting that these two regulators are not essential for regulation of cellulase biosynthesis under the conditions used in this work.

**Figure 8:**
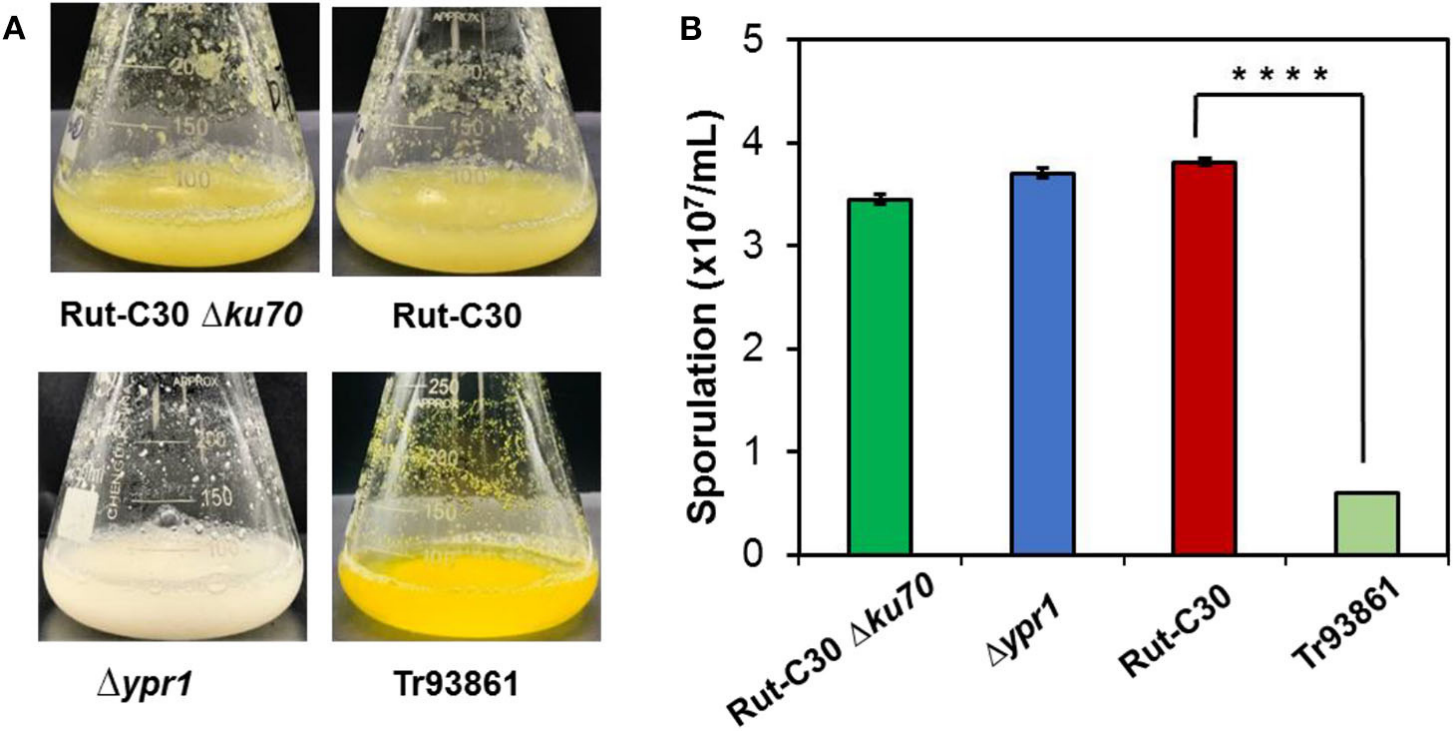
Effects of *ypr1* on pigment production and sporulation. *T. reesei* Tr93861 is the mutant strain overexpressing *ypr1* in *T. reesei* Rut-C30, whereas strain Δ*ypr1* was obtained by deleting *ypr1* in *T. reesei* Δ*ku70*. The mutant strains and their respective parental strains were cultured on cellulase production medium for 5 days, and pigment production was shown **(A)**. The sporulation assay of the *T. reesei* strains was implemented when cultured on MEA for 7 days **(B)**. Asterisks indicate significant differences from reference strains (****p < 0.001, Student's *t*-test).

In terms of cell morphology, the phenotype of *T. reesei* Δ*74374* had no obvious change compared with the parental strain, and the fermentation broth was pale yellow under the condition of cellulose medium. *T. reesei* Δ*ypr1* recovered the similar level of spore-forming ability to the parental strain, so did the growth biomass and mycelial morphology. During the cellulase fermentation process, the Δ*ypr1* strain no longer secreted yellow pigment, and the fermentation broth became white in contrast to Rut-C30. However, the yellow pigment production in the *ypr1* overexpression strain Tr93861 enhanced significantly. Meanwhile, conidiation of Tr93861 strain decreased remarkably (Figure 8). Hypersecretion of the yellow pigment may be the direct cause of significant reduction in vegetative growth owing to the metabolic burden or the growth limiting effect of yellow pigment (Derntl et al., 2017a). The Ypr1 homolog in Rut-C30 (663 aa) have four regions of amino acid residues (insertions and deletions) in the C-terminal part of middle homology region (MHR) and regulatory region compared that of Ypr1 in QM6a (gene ID: 102499, 686 aa), which maybe resulted in the different regulatory profile for cellulase production and pigment secretion between Rut-C30 and QM6a (Figure S6).

To further analyze the effect of *ypr1* knockout on the secretion of *T. reesei* cellulase, comparative transcriptome analysis was performed for the knockout mutant *T. reesei* Δ*ypr1* and the parental strain Rut-C30 Δ*ku70* in MA medium with glucose as carbon source. Unexpectedly, we found that the transcription of the glycoside hydrolase genes in *T. reesei* Δ*ypr1* mutant were significantly changed compared to the parental strain *T. reesei* Rut-C30 Δ*ku70*. Table 2 lists transcription changes in several major glycoside hydrolases and transcriptional regulator genes. The transcriptional quantity of major cellulases has been improved remarkably in *T. reesei* Δ*ypr1*, the minimum change fold is 1-fold increased (*cbh1 gene*), and the maximum transcript level change happened to the *eg1* gene which increased by 3.8 times. The transcriptional levels for most hemicellulase genes are improved by at least 1.35-fold. Meanwhile, the transcriptions of *cel61a, cip1* genes encoding the auxiliary proteins, and *axe1* gene (encoding acetylxylan esterase) have increased by 0.8, 1.6 and 2.4-fold, respectively, whose positions are located in close proximity to the gene *ypr1* locus (scaffold_1:507183-509547), which means that the deletion of Ypr1 protein facilitates the transcription of the surrounding glycoside hydrolase genes. Meanwhile, the transcripts of genes encoding the major cellulase regulatory factors, such as the genes of Xyr1 and Ace3, have increased significantly, except for Vib1.

**Table 2:** Changes of major glycoside hydrolases and transcription factors in *T. reesei* Δ*ypr1* comparing with that of the control strain.

**GeneID**	**Protein**	Log_**2**_FC^*^
125125	CBH1	0.99
122470	CBG2	1.78
5304	EG1	2.27
72489	EG2	1.69
124438	EG3	1.71
25940	EG5	1.06
136547	BGL1	1.52
38418	XYN1	1.89
124931	XYN2	0.31
23616	XYN3	1.38
90847	XYN4	1.23
134945	XYN5	1.31
140746	BXL1	1.10
139633	CEL61a	0.87
122518	CEL61b	0.42
104220	SWO1	1.42
121449	CIP1	1.40
125575	CIP2	0.68
98455	ACE3	1.18
98788	XYR1	1.58
31634	YPR2	−7.55
125610	Vib1	0.41

* *FC, Fold change of the transcription of genes in T. reesei Δypr1 over that in T. reesei Rut-C30 Δku70*.

## Discussion

In our previous work, *T. reesei* strains overexpressing *AZFP-U3* (Zhang et al., 2016) and *AZFP-M2* (Meng et al., 2020) showed enhanced cellulase production. Comparing with these two AZFP genes, *AZFP-U5* has different sequence and also showed different regulatory patterns on cellulase biosynthesis. The strain overexpressing *AZFP-U5* showed the best phenotypes among the three AZFP strains, with higher protein secretion level and higher activities of various cellulases and xylanases. Therefore, it is of great interest to further explore the mechanisms by which this AZFP functions.

In the previous studies, transcriptome analysis was performed using *T. reesei* grown in different carbon source (glucose, lactose, sophorose, or cellulose) and lignocellulose substrates, and data analysis has been focused on the functions of known major regulators, such as Xyr1 and Cre1 (dos Santos Castro et al., 2014, 2016; Hakkinen et al., 2014). Nevertheless, detailed analysis of global transcription as well as metabolic pathways for cellulase production in *T. reesei* is still lacking. In this work, we present extensive and comprehensive analysis of global transcription network reprogramming by the artificial regulator AZFP-U5, and assume that multiple pathways may be involved in cellulase biosynthesis by the function of AZFP-U5. For example, AZFP-U5 activates the transcription of glycoside hydrolase genes in the mRNA regulatory pathway, and also leads to changes of a series of metabolic processes, including mRNA synthesis, processing, and degradation, which may then lead to an increase in protein translation, processing, and secretion. Meanwhile, activation of the protein degradation pathway may be achieved by accumulation of unfolded proteins. The improvement of tRNA synthetase genes in the aminoacyl-tRNA synthesis pathway further indicated that the protein synthesis in *T. reesei* U5 were more active compared to that in *T. reesei* Rut-C30. In the reprogramming of global gene transcription, *T. reesei* U5 strain may be supplied with more energy, reducing power, and protein synthesis precursors by the activated intercellular metabolic pathways, which was also reflected from the comparative transcriptome results. After investigating dynamic changes of global transcription, we found different transcription profiles at 24 and 48 h. At 48 h, genes encoding marker proteins in the endoplasmic reticulum still have an up-regulation in *T. reesei* U5, indicating that AZPF-U5 may also have stimulating effects on protein processing, traffickling, and secretion pathway.

**Figure 9:**
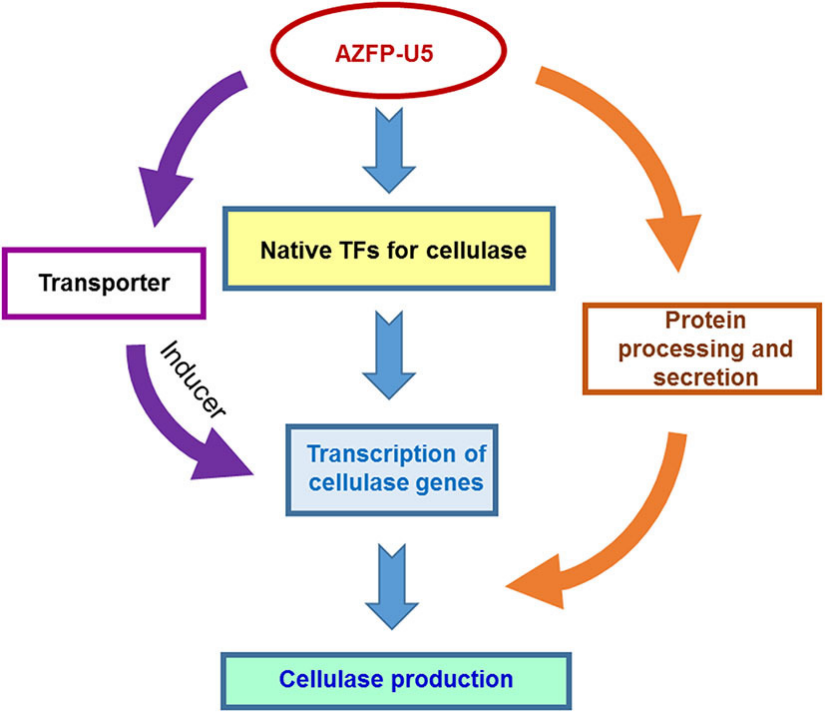
Proposed model for the regulatory effect of AZF-U5 on cellulase synthesis. TFs transcription factors.

In addition to changes in the key metabolic processes, variation of transcription in several sugar transporters were discovered. AZFP-U5 may activate transcription of major transporter genes, which could transport potential carbon source inducers or transmit the carbon signal for induction, followed by transcriptional activation of the glycoside hydrolase genes regulated by AZFP-U5, directly, or indirectly. Meanwhile, the AZFP-U5 activates the stress response pathway and the transcription of the peroxisome protein genes, indicating that this artificial regulator has a potential positive effect on the release of reactive oxygen and external environmental stress response in protein and peptide synthesis. The possible mechanism by which AZFP-U5 works on cellulase production is shown in Figure 9. Further studies are needed to explore the directly regulated genes by AZFP-U5 as well as key genes on regulation of glycoside hydrolase genes.

During the transcriptome analysis, many putative regulator genes showed enhanced transcription by AZFP-U5 overexpression, of which 15 were selected for further investigation. However, the results did not show significant increase in cellulase production by overexpressing these regulators. It may be possible that individual expression of these regulators may not be sufficient to enhance cellulase biosynthesis, and the advantage of AZFP-U5 is that it simultaneously regulates multiple gene transcription by binding with various target genes, and induce changes in related signal transduction and metabolic pathways. It will be interesting to test the combinatory effects of the regulators for regulating cellulase production in the future work.

In this study, we found that Ypr1 in *T. reesei* Rut-C30 not only regulates the formation of secondary metabolites, but also participates in the transcriptional regulation of glycoside hydrolase genes when glucose was used as a carbon source. From transcriptome analysis, deletion of Ypr1 significantly increased the transcription of extracellular glycoside hydrolases and major transcription factor genes. We speculate that the secondary metabolites have a coupling effect with the cellulase production. This crosslink is also reflected in the transcription factor Xpp1 in *T. reesei*, the presence of Xpp1 can inhibit the synthesis of hemicellulase genes and inhibit the production of secondary metabolites in the cell, which acts as a switch function between the primary and secondary metabolism (Derntl et al., 2017b). Moreover, *T. reesei* ZC121 with up-regulated Ypr1 could overproduce the yellow pigment, but almost produce no extracellular cellulase (Li et al., 2018).

Ypr2 is a transcription factor that affects the production of yellow pigment, which has an inhibition effect on Ypr1 expression (Derntl et al., 2016). Deletion of Ypr1 also severely affected the transcription of Ypr2 (almost no ypr2 transcript in *T. reesei* Δ*ypr1* strain). Moreover, under dark conditions, Ypr2 (gene ID: 102497 in *T. reesei* QM6a, 664 aa) can affect the transcription of genes regulated by Cre1 in *T. reesei* QM6a, and Ypr2 also could regulate Ypr1 (Hitzenhammer et al., 2019). However, the Ypr2 protein in *T. reesei* Rut-C30 (gene ID: *TrC30_31634*, 511 aa) contains a zinc finger-free DNA binding domain in *T. reesei* Rut-C30 (the first 175 amino acid residues missed), which will cause severe impact on DNA binding and regulatory function. Therefore, the specific role and function of Ypr2 lacking the binding domain in metabolism and pigment secretion requires further analysis (Figure S7).

The results in this study showed possible cross talk between cellulase production and pigment secretion in *T. reesei* regulated by the major regulator Ypr1 protein. Meanwhile, deletion of Ypr1 significantly increase the transcription of major glycoside hydrolase and transcription factor genes. Actually, CAZyme genes in *T. reesei* are non-randomly distributed within the genome, about (41%) CAZyme genes are found in 25 discrete regions containing specific regulators or secondary metabolites clusters (Martinez et al., 2008; Hakkinen et al., 2014). Ypr1 not only locates into nearly adjacent region of glycoside hydrolases, but also possess the co-regulated effect for *TrC30_69551* and some glycoside hydrolase genes, including *axe1, cip1*, and *cel61a*. However, there is no significant change in Δ*ypr1* for extracellular cellulase secretion compared to that from parental strain when cellulose or glucose is used as the substrate. In *T. reesei* Δ*ypr1*, the transcript level of *ypr2* nearly disappeared (Log_2_FC = −7.5), which may intensify CCR effect on cellulase translation and secretion. Another reason may be the existence of complex post-transcriptional or post-translational mechanisms for cellulase proteins, which need to be revealed in further studies.

## Conclusion

*T. reesei* mutant U5 bearing an AZFP (AZFP-U5) was focused, which secretes more proteins and showed significantly higher cellulase and xylanase production than its parental strain *T. reesei* Rut-C30. Comparative transcriptome analysis showed enhanced transcription and post-translational modifications of glycoside hydrolases by overexpression of AZFP-U5. Furthermore, deletion of *ypr1* and *TrC30_74374* did not affect cellulase production. Deletion of *ypr1* affected cellulase gene transcription, suggesting possible crosslink between pigment production and cellulase gene expression by the AZFP-U5-associated regulator Ypr1.

## Data Availability Statement

The raw data supporting the conclusions of this article will be made available by the authors, without undue reservation, to any qualified researcher.

## Author Contributions

FZ and X-QZ designed the experiments. FZ and J-XL performed the experiments as well as data analysis, and also prepared the draft of the manuscript. VC, C-GL, and F-WB participated in revision of the manuscript. X-QZ supervised the experiments and critically revise the manuscript. All authors contributed to the article and approved the submitted version.

## Conflict of Interest

The authors declare that the research was conducted in the absence of any commercial or financial relationships that could be construed as a potential conflict of interest.
