# Supplementary material for: Global Reprogramming of Gene Transcription in Trichoderma reesei by Overexpressing an Artificial Transcription Factor for Improved Cellulase Production and Identification of Ypr1 as an Associated Regulator
Source: Front Bioeng Biotechnol. 2020 Jul 3;8:649. doi: 10.3389/fbioe.2020.00649 (PMC7351519; doi:10.3389/fbioe.2020.00649)
Supplement: Supplementary file 1 [file Table_1.docx]

**Supplementary materials for the manuscript by Zhang et al., 2020**

**Supplementary tables**

**Table S1** Primers used for *T. reesei* strain construction

| **Primers** | **Sequence (5' to 3')** |
| --- | --- |
| **Cloning, construction and verification (overexpression of regulators) of *T. reesei* strains**  **and verification**  **(overexpression of regulators)** | |
| pCB303-U5F | CCGGAAGAACCCTCTTAACCATGGGCGCCCCCCCCAAG |
| pCB303-U5R | AACGTTAAGTGGATCCTCTAGGCTGGCGTAGTCGGGGACGTC |
| 23164F | CGCAGCTACAGCACAACCATGGACGTTTCCACTGTGCCCTGCAC |
| 23164R | TAACGTTAAGTGTATTCTCTATACCACGCCAAGGACTGGTGG |
| 23425F | CGCAGCTACAGCACAACCATGTCGACGGCTAAAGCGACCAAG |
| 23425R | TAACGTTAAGTGTATTCTCTAAAACGAGTCCTGATTTTCCGTA |
| 141745F | CGCAGCTACAGCACAACCATGACCTCGCTGTACGACCAGCTA |
| 141745R | TAACGTTAAGTGTATTCTCTATTGCACCGGAATAGATGTATC |
| 102019F | CGCAGCTACAGCACAACCATGAAGAAATCAATTGCTTGCGGCG |
| 102019R | TAACGTTAAGTGTATTCTCTACCATCCTGATACCTCAACGTTTC |
| 129764F | CGCAGCTACAGCACAACCATGACGGCCCTGCTCAGGTCCACG |
| 129764R | TAACGTTAAGTGTATTCTCTACTTCTTTTCAACGTTTCCAACAC |
| 38417F | CGCAGCTACAGCACAACCATGTTCCGCAGGCAGGTTCCGCCAGCCA |
| 38417R | TAACGTTAAGTGTATTCTCTATGCTTTTACAAGCTGCTCCTCTATCC |
| 124902F | CGCAGCTACAGCACAACCATGAGCTACTCTCTCTACGACGCCACCG |
| 124902R | TAACGTTAAGTGTATTCTCTACACCTTGTTGGTCAGGAACTCGCCGA |
| 81999F | CGCAGCTACAGCACAACCATGGTAGCACATAGTCTACCCTCTCCTC |
| 81999R | TAACGTTAAGTGTATTCTCTATATCGGCACCATGTCGACGTCGAAG |
| 38522F | CGCAGCTACAGCACAACCATGGAGCGACAAACGGATCTTAGGGTC |
| 38522R | TAACGTTAAGTGTATTCTCTATGCTAGGGTCGCCGGAGGCATTGTACT |
| 99728F | CGCAGCTACAGCACAACCATGACCATGCATCACCAGCTTAA |
| 99728R | TAACGTTAAGTGTATTCTCTAGTCAGTTTCCTCTTCAATCACCT |
| 93160F | CGCAGCTACAGCACAACCATGAGTCGATCGTCCGTGCATCAGAATGC |
| 93160R | TAACGTTAAGTGTATTCTCTACATGTCCTCAACAAGCCACTGCTGCATC |
| 26551F | CGCAGCTACAGCACAACCATGGCCACCGTCTTGAAAAACGTTGCCCT |
| 26551R | TAACGTTAAGTGTATTCTCTAGTCGCTGAGTGTCTTCTTGACAATTGC |
| 72675F | CGCAGCTACAGCACAACCATGGACGGGCGATATGAAAGCCTGGCGAC |
| 72675R | TAACGTTAAGTGTATTCTCTACCTCATTGCTTGCATGATTTCGTGCCGC |
| 93861F | CGCAGCTACAGCACAACCATGGGGAGCAGCGCCACGGCCACCACGACT |
| 93861R | TAACGTTAAGTGTATTCTCTATGACCAGGAGCCATCGCCCTGATCGAAT |
| 74374F | CGCAGCTACAGCACAACCATGGTTGGAGAACGCCGTGCTTGTGACGCT |
| 74374R | TAACGTTAAGTGTATTCTCTATTGCCCTATGGCACCATTCTCCAGCCCT |
| **Cloning and construction** **(knockout cassette for putative regulators)** | |
| hphCAS-F | GCACTATTGATCATCCGATAGCTCTG |
| hphCAS-R | ATCCCCCCAAACTACAGCGCCGCTG |
| For 2000-bp upstream region of *TrC30_93861* | |
| 93861U-F | TGTTGGTTGGCAAGAAGATGAACCATG |
| 93861U-o-hph-R | AGCTATCGGATGATCAATAGTGCAACGGTAGGATAATGAGGCAAGATG |
| For 2000-bp downstream region of *TrC30_93861* | |
| 93861D-o-hph-F | AGCGGCGCTGTAGTTTGGGGGGATACAGCAGAATCAGAGCAAATCTCCCATC |
| 93861D-R | GCGTCTAGAACGTCAAATGGCCCGTCG |
| For 2000-bp upstream region of *TrC30_74374* | |
| 74374U-F | TTCGTGAATGGCTGCATCGGCACGGAC |
| 74374U-o-hph-R | AGCTATCGGATGATCAATAGTGCACCTCCTCACCTGCTGCGTCTTGTC |
| For 2000-bp downstream region of *TrC30_74374* | |
| 74374D-o-hph-F | AGCGGCGCTGTAGTTTGGGGGGATACTTCTGTGACTGTATACGCATGTTGG |
| 74374D-R | CCACCCAGTCAGCACTAACATCATAG |
| **Genomic characterization for regulatory gene deletion mutants** | |
| 93861VDelF | ATGGGGAGCAGCGCCACGGCCACCACG |
| 93861VDelR | TTGAGGCGACATGGCCGACGCAATGGTCC |
| 74374VDelF | ATGGTTGGAGAACGCCGTGCTTGTGACGC |
| 74374VDelR | CCTGTCTGAGGCTGCGTCGTCTGGTTGTTG |
| hphCasDV-F | GTGCGCCAGGTGAGAGTATGTCTCGGGAGG |
| hphCasUV-R | AGCCGTGCCGAAATCCTTACAGCTTGTGTTG |
| 93861Useq-F | ACATGTGCCCCATCTTCATGCAGTGCC |
| 93861Dseq-R | ATAGTCCCAGACCACCGGCTCCAGTGG |
| 74374Useq-F | AATGATGGACACTCGCTTTCGTTCTCCACC |
| 74373Dseq-R | TGTCAACAGCCATCTGTATAAATAAACCAGC |

**Table S2** Primers used in this study for RT-qPCR, qPCR and TAIL-PCR analysis

| **Usage and primer name** | **Sequence (5' to 3')** |
| --- | --- |
| **qPCR analysis** |  |
| AZFPU5-F | GGCCTTCTCCGTCAGCTCCAGCC |
| AZFPU5-R | TGGCGCTTCTTGTGGCGGTTGAG |
| hphqPCR-F  hphqPCR-R | CCGACGTGCCTTATCTGCCTTC  CGCCTGTCCAATGTCCAGTTCC |
| **RT-qPCR analysis** |  |
| cbh1-F | ACGAGTTCTCTTTCGATGTTGATG |
| cbh1-R | CGGTGTTGGTGGGATACTTG |
| cbh2-F | TCCTGGTTATTGAGCCTGAC |
| cbh2-R | GCAACATTTGGAAGGTTCAG |
| eg1-F | CTCAGATGGACGAGAACGGG |
| eg1-R | CTGGTGGCTAGTGTTGAGGG |
| eg2-F | AACAAGTCCGTGGCTCCATT |
| eg2-R | TCCGCTCCAACCAATACCTC |
| bgl1-F | CCGAGTGATCTGTTCCAGAATGT |
| bgl1-R | CTGGGTGCTGAAGATGGGTAG |
| xyn1-F | AAACTACCAAACTGGCGG |
| xyn1-R | TTGATGGGAGCAGAAGATCC |
| xyn2-F | CGGCTACTTCTACTCGTACTG |
| xyn2-R | TTGATGACCTTGTTCTTGGTG |
| eg4-F | TCAACTACATCATCCCTGGACCT |
| eg4-R | CCGTTGTCGTGGTTCTGCT |
| xyr1-F | CCATCAACCTTCTAGACGAC |
| xyr1-R | AACCCTGCAGGAGATAGAC |
| bglR-F | GCAAGGTCAAGTGCGATGG |
| bglR-R | CTGTTGATGCGGTTGTGGA |
| ace1-F | ACCAAGACCAACGGCAAGA |
| ace1-R | CGTGGAGGAAGGCGTAGACA |
| ace2-F | GCCTCAATGCTGCTCTCTGTT |
| ace2-R | GACGAACGACCTTTGCTTCTCT |
| tef1-F | CTGGGTGTCAAGCAGCTCA |
| tef1-R | GAGATGGGGACGAAAGCAAC |
| 69465-F | CGTCTTCTGGAGCTCTACCG |
| 69465-R | GAGTCGCTGAGCTTCTGGTC |
| 98900-F | AGAAGGAAATGGCCCTGATT |
| 98900-R | ACATGAGGGCCTGGTTGTAG |
| 123550-F | ATGTCCCCTTCTGCAATCAG |
| 123550-R | GTGGTCATAGCCGGTGAAGT |
| 133544-F | CATGCCCTCAAGACTCTTCC |
| 133544-R | TCACCAAGGCCATAGACCTC |
| 136048-F | GCGTGTCTACCCGTTCTACC |
| 136048-R | CAGTAGCAGACGTGGACGAA |
| **TAIL-PCR analysis** |  |
| U5RB-1R | GCATGGTTGCCTAGTGAATGCTCCGT |
| U5RB-2R | CGTTTACCCAGAATGCACAGGTACAC |
| U5RB-3R | CACATCTCCACTCGACCTGCAGGCAT |
| AD1 | ACGATGGACTCCAGAGCGGCCGCVNVNNNGGAA |
| AD2 | ACGATGGACTCCAGAGCGGCCGCBNBNNNGGTT |
| AD3 | ACGATGGACTCCAGAGCGGCCGCVVNVNNNCCAA |
| AD4 | ACGATGGACTCCAGAGCGGCCGCBDNBNNNCGGT |

**Table S3** The nucleotide sequence and protein sequence of regulator AZFP-U5

| **> *azfp-u5* nucleotide sequence** |
| --- |
| ATGGGCGCCCCCCCCAAGAAGAAGCGCAAGGTCGCCGGCTCCACCAGCAACGGCCGCCAGTGCGCCGGCATCCCCGGCGAGAAGCCCTACACCTGCAAGCAGTGCGGCAAGGCCTTCTCCGTCAGCTCCAGCCTGCGCCGCCACGAGACCACCCACACCGGCGAGAAGCCCTACGTCTGCGACGTCGAGGGCTGCACCTGGAAGTTCGCCCGCAGCGACGAGCTCAACCGCCACAAGAAGCGCCACACGGGCGAGAAGCCCTACCGCTGCAAGTACTGCGACCGCTCCTTCAGCGACTCCAGCAACCTGCAGCGCCACGTCCGCAACATTCATACGGGTGAGAAGCCCTACACGTGCTCCTACTGCGGCAAGTCCTTCACCCAGAGCAACACCCTCAAGCAGCACACCCGCATCCACACCGGCGAGAAGGCCGCCGCCGCCAACTTCAACCAGTCCGGCAACATCGCCGACTCCAGCCTGAGCTTCACCTTCACCAACTCCAGCAACGGCCCCAACCTCATCACCACCCAGACCAACTCCCAGGCCCTGAGCCAGCCCATCGCCTCCAGCAACGTCCACGACAACTTCATGAACAACGAGATCACCGCCAGCAAGATCGACGACGGCAACAACTCCAAGCCCCTCAGCCCCGGCTGGACCGACCAGACCGCCTACAACGCCTTCGGCATCACCACCGGCATGTTCAACACCACCACCATGGACGACGTCTACAACTACCTGTTCGACGACGAGGACACCCCCCCCAACCCCAAGAAGGAGATCTCCATGGCCTACCCCTACGACGTCCCCGACTACGCCAGCTAA |

| **>AZFP-U5 protein sequence** |
| --- |
| MGAPPKKKRKVAGSTSNGRQCAGIPGEKPYTCKQCGKAFSVSSSLRRHETTHTGEKPYVCDVEGCTWKFARSDELNRHKKRHTGEKPYRCKYCDRSFSDSSNLQRHVRNIHTGEKPYTCSYCGKSFTQSNTLKQHTRIHTGEKAAAANFNQSGNIADSSLSFTFTNSSNGPNLITTQTNSQALSQPIASSNVHDNFMNNEITASKIDDGNNSKPLSPGWTDQTAYNAFGITTGMFNTTTMDDVYNYLFDDEDTPPNPKKEISMAYPYDVPDYAS* |

**Table S4** Functional distributions of differentially expressed genes in *T. reesei* U5 comparing with its parental strain by KEGG and KOG databases.

| **Gene function catalogs** | **24 h** | **48 h** |
| --- | --- | --- |
| Cell defense | 0.67% | 1.21% |
| Development (cell cycle) | 7.56% | 5.32% |
| DNA replication, precursor, repairs | 8.40% | 4.92% |
| Energy | 4.35% | 4.68% |
| Function unknown | 20.58% | 29.35% |
| CAZymes | 2.74% | 7.10% |
| Metabolism | 20.84% | 18.95% |
| Protein fate (translation, folding, modification, sorting and secretion) | 12.41% | 7.66% |
| RNA synthesis, processing, modification, surveillance and degradation | 6.09% | 4.11% |
| Secondary metabolism | 6.19% | 8.79% |
| Signal transduction | 4.95% | 4.03% |
| Transcription | 5.22% | 3.87% |

**List of additional information provided in separate Excel files**

**Additional file 1.** Expression profile of genes potentially regulated by AZFP-U5

**Additional file 2.** Differentially expressed genes (DEGs) between *T. reesei* U5 and Rut-C30

**Additional file 3.** DEGs of transcription factors between *T. reesei* U5 and Rut-C30

**Additional file 4.** DEGs of CAZymes between *T. reesei* U5 and Rut-C30

**Additional file 5.** DEGs related to protein processing and secretion between *T. reesei* U5 and Rut-C30

**Figure legends for supplementary data**

**Figure S1** Cellulase activities between the mutant *T. reesei* U5 and Rut-C30

(A) CMCanase activities; (B) β-glusosidase activities; (C) Xylanase activities. The activities were determined at 7th day’s fermentation for *T. reesei* U5 and Rut-C30.

**Figure S2** Verification for transcriptomics data of *T. reesei* U5 and Rut-C30 by RT-qPCR

**Figure S3** Schematic drawing of deletion of putative regulators in *T. reesei* Rut-C30 (A: ∆*ypr1*::hph; B: ∆*74374*::hph) and representative electrophoresis results of the corresponding PCR products for positive transformants (C)

**Figure S4** The copy number of *hph* deletion cassette in ∆*93861* (∆*ypr1*) and ∆*74374* strains

**Figure S5** FPase activities of *T. reesei* ∆*93861* (∆*ypr1*) and ∆*74374* strains

The strains were cultured in MA medium with 2% cellulose and 2% wheat bran as carbon sources for 7 d.

**Figure S6** Ypr1 (TrC30_93861) protein sequence alignment in Rut-C30 and QM6a

**Figure S7** Ypr2 (TrC30_31647) protein sequence alignment in Rut-C30 and QM6a

**
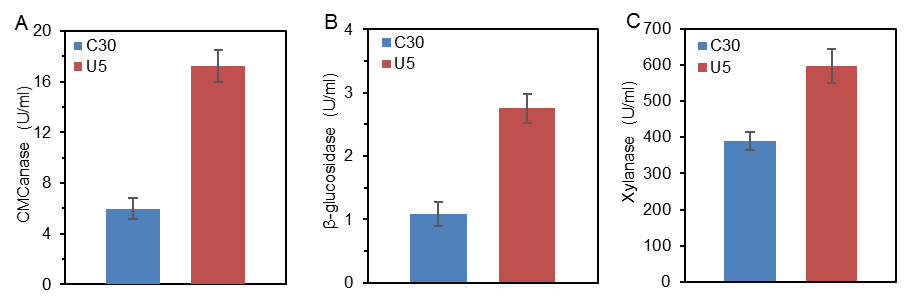
**

**Figure S1**


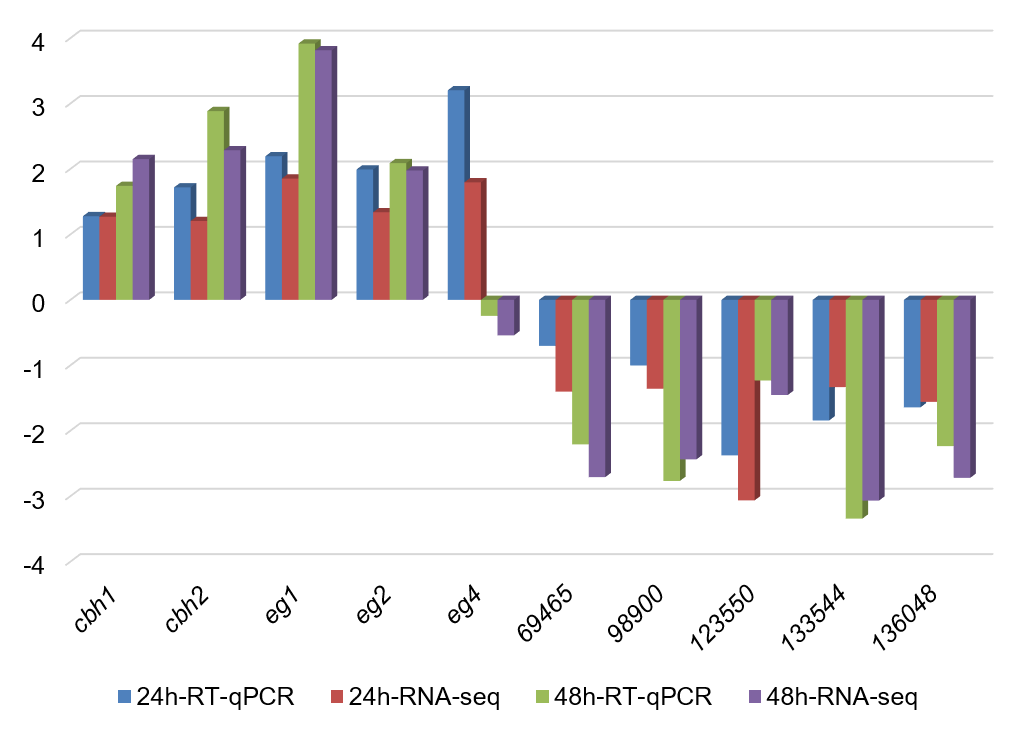


**Figure S2**

**
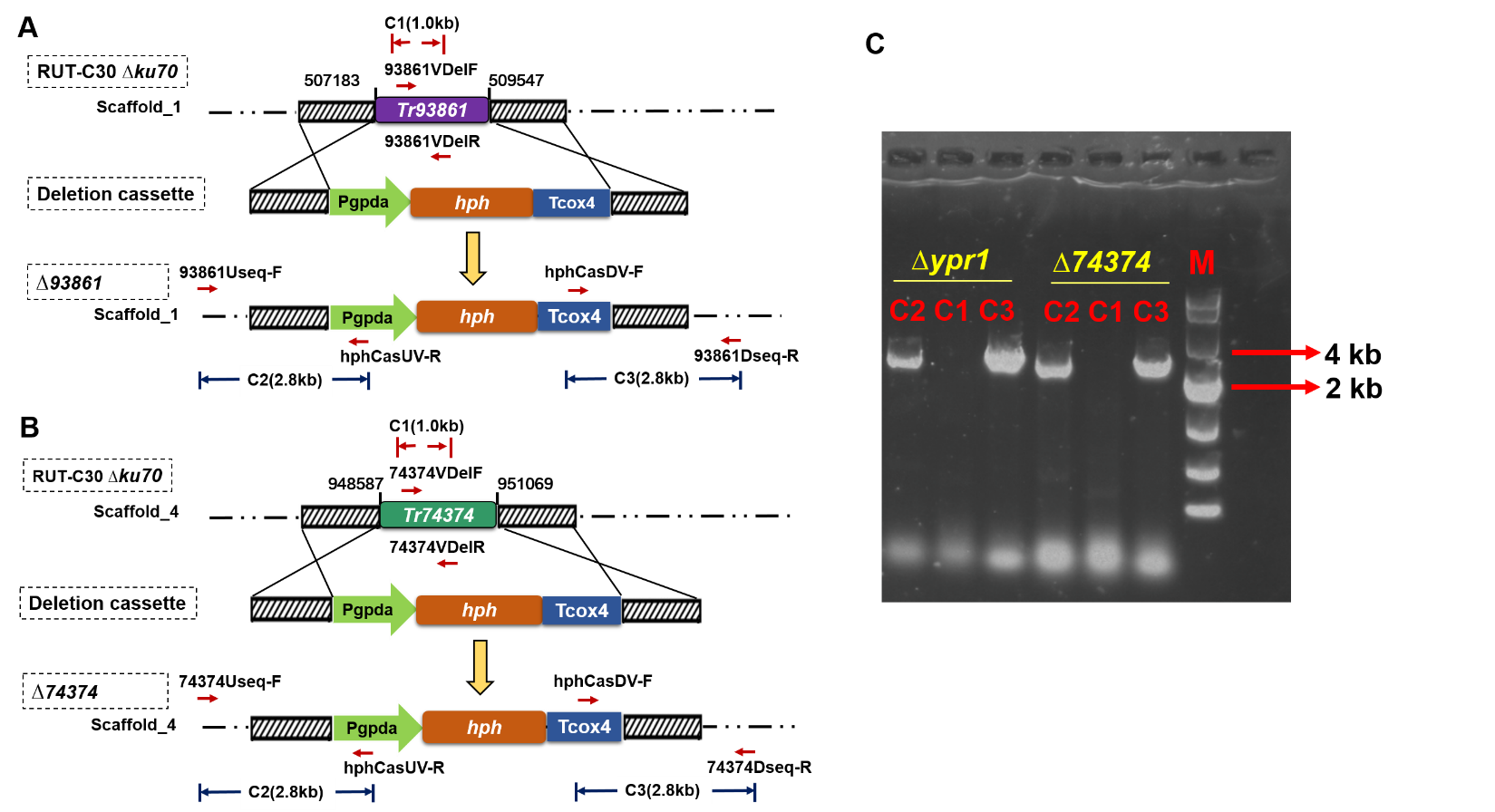
**

**Figure S3**

**
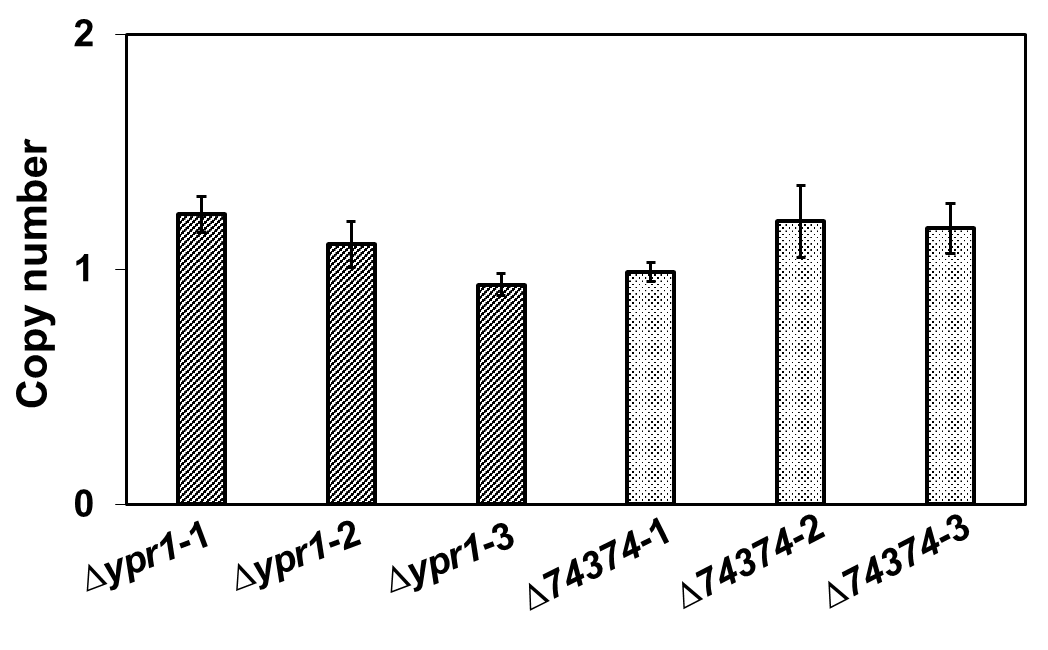
**

**Figure S4**


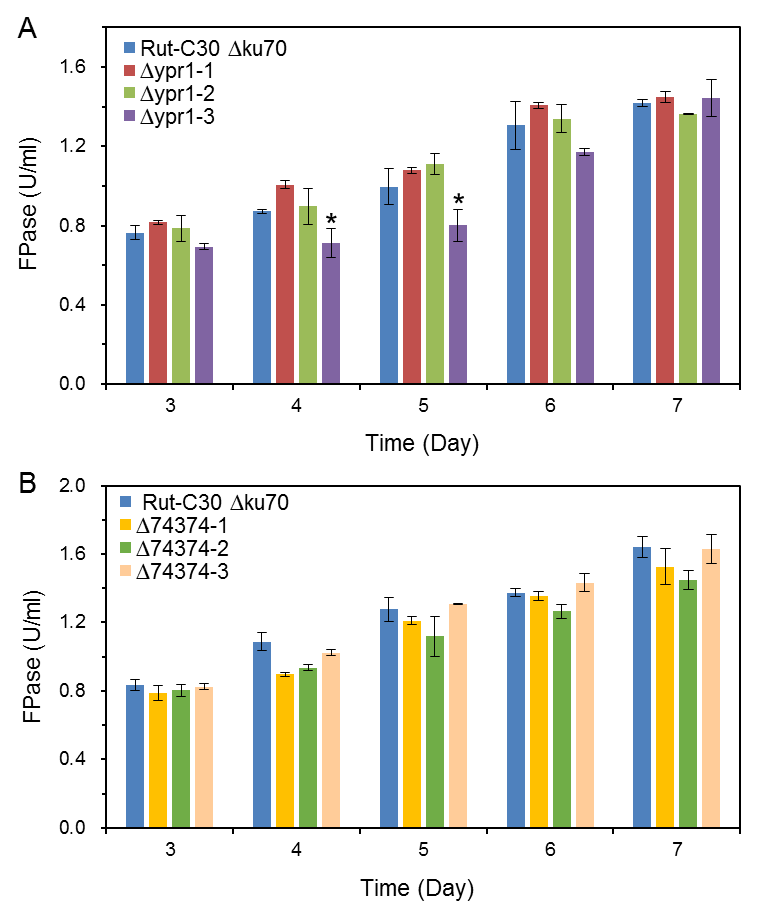


**Figure S5**

**
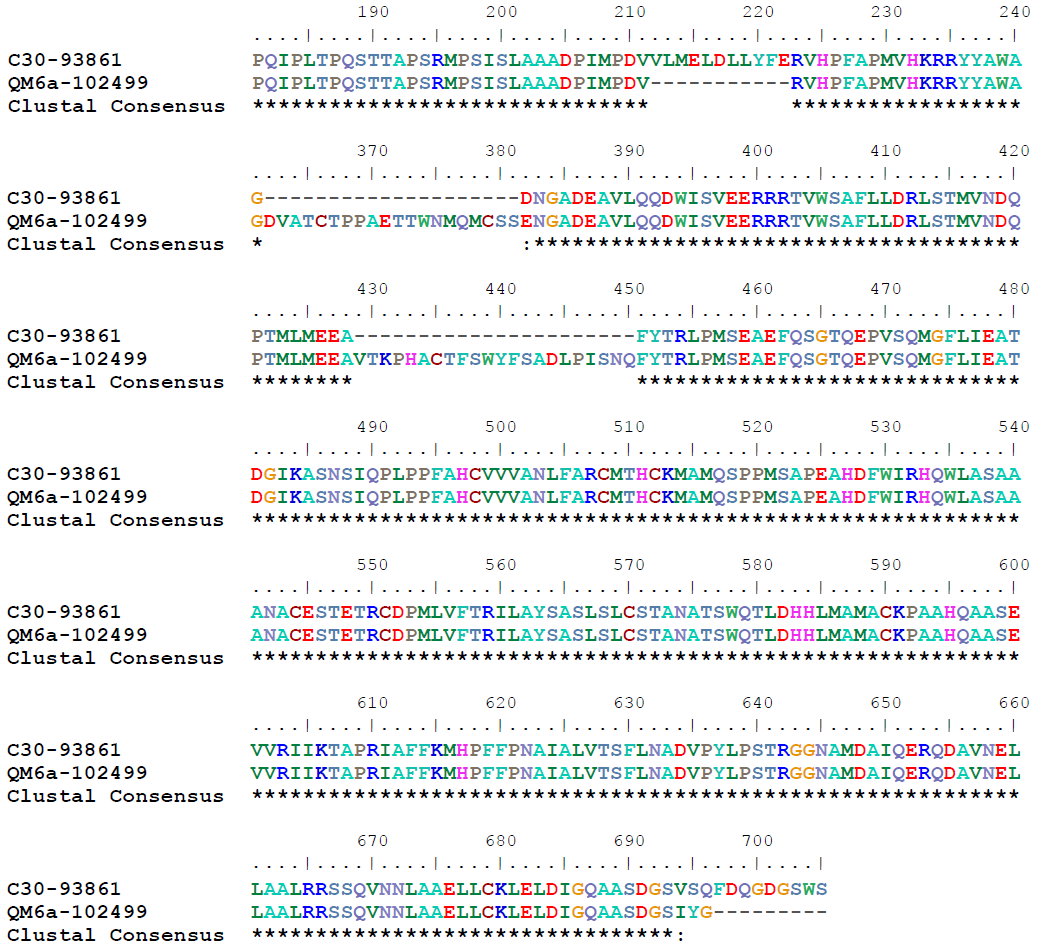
**

**Figure S6**

**
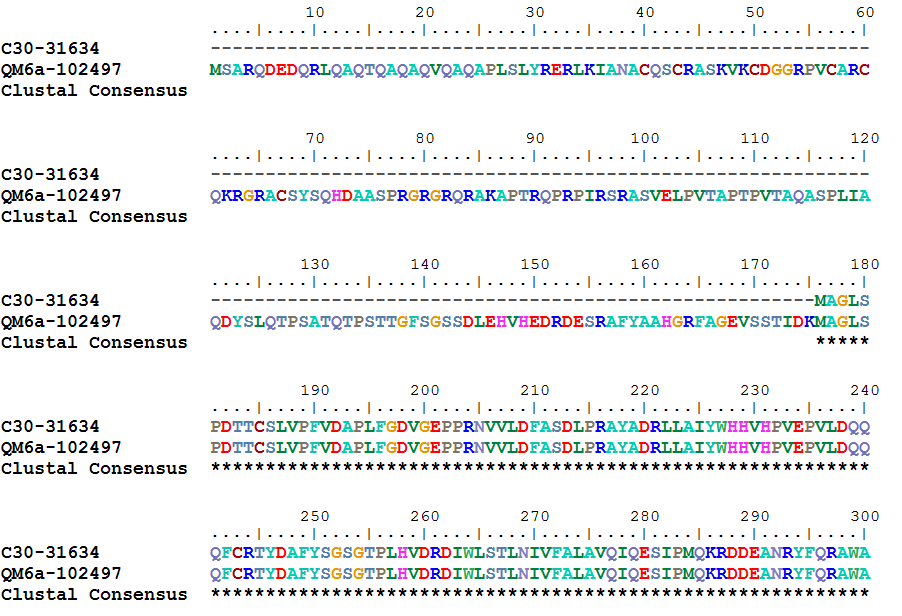
**

**Figure S7**
